# Dimensionality-driven metal to Mott insulator transition in two-dimensional 1T-TaSe_2_

**DOI:** 10.1093/nsr/nwad144

**Published:** 2023-05-16

**Authors:** Ning Tian, Zhe Huang, Bo Gyu Jang, Shuaifei Guo, Ya-Jun Yan, Jingjing Gao, Yijun Yu, Jinwoong Hwang, Cenyao Tang, Meixiao Wang, Xuan Luo, Yu Ping Sun, Zhongkai Liu, Dong-Lai Feng, Xianhui Chen, Sung-Kwan Mo, Minjae Kim, Young-Woo Son, Dawei Shen, Wei Ruan, Yuanbo Zhang

**Affiliations:** State Key Laboratory of Surface Physics, New Cornerstone Science Laboratory, and Department of Physics, Fudan University, Shanghai 200438, China; Shanghai Qi Zhi Institute, Shanghai 200232, China; Shanghai Research Center for Quantum Sciences, Shanghai 201315, China; Institute for Nanoelectronic Devices and Quantum Computing, Fudan University, Shanghai 200433, China; Zhangjiang Fudan International Innovation Center, Fudan University, Shanghai 201210, China; State Key Laboratory of Functional Materials for Informatics, Shanghai Institute of Microsystem and Information Technology (SIMIT), Chinese Academy of Sciences, Shanghai 200050, China; School of Physical Science and Technology, ShanghaiTech University, Shanghai 201210, China; Korea Institute for Advanced Study, Seoul 02455, South Korea; State Key Laboratory of Surface Physics, New Cornerstone Science Laboratory, and Department of Physics, Fudan University, Shanghai 200438, China; Shanghai Qi Zhi Institute, Shanghai 200232, China; Shanghai Research Center for Quantum Sciences, Shanghai 201315, China; Institute for Nanoelectronic Devices and Quantum Computing, Fudan University, Shanghai 200433, China; Zhangjiang Fudan International Innovation Center, Fudan University, Shanghai 201210, China; School of Emerging Technology and Department of Physics, University of Science and Technology of China, Hefei 230026, China; State Key Laboratory of Surface Physics, New Cornerstone Science Laboratory, and Department of Physics, Fudan University, Shanghai 200438, China; Shanghai Qi Zhi Institute, Shanghai 200232, China; Shanghai Research Center for Quantum Sciences, Shanghai 201315, China; Institute for Nanoelectronic Devices and Quantum Computing, Fudan University, Shanghai 200433, China; Zhangjiang Fudan International Innovation Center, Fudan University, Shanghai 201210, China; State Key Laboratory of Surface Physics, New Cornerstone Science Laboratory, and Department of Physics, Fudan University, Shanghai 200438, China; Shanghai Qi Zhi Institute, Shanghai 200232, China; Shanghai Research Center for Quantum Sciences, Shanghai 201315, China; Institute for Nanoelectronic Devices and Quantum Computing, Fudan University, Shanghai 200433, China; Zhangjiang Fudan International Innovation Center, Fudan University, Shanghai 201210, China; Advanced Light Source, Lawrence Berkeley National Laboratory, Berkeley, CA 94720, USA; State Key Laboratory of Surface Physics, New Cornerstone Science Laboratory, and Department of Physics, Fudan University, Shanghai 200438, China; Shanghai Qi Zhi Institute, Shanghai 200232, China; Shanghai Research Center for Quantum Sciences, Shanghai 201315, China; Institute for Nanoelectronic Devices and Quantum Computing, Fudan University, Shanghai 200433, China; Zhangjiang Fudan International Innovation Center, Fudan University, Shanghai 201210, China; School of Physical Science and Technology, ShanghaiTech University, Shanghai 201210, China; ShanghaiTech Laboratory for Topological Physics, Shanghai 200031, China; Key Laboratory of Materials Physics, Institute of Solid State Physics, Hefei Institutes of Physical Science, Chinese Academy of Sciences, Hefei 230031, China; Key Laboratory of Materials Physics, Institute of Solid State Physics, Hefei Institutes of Physical Science, Chinese Academy of Sciences, Hefei 230031, China; High Magnetic Field Laboratory, Hefei Institutes of Physical Science, Chinese Academy of Sciences, Hefei 230031, China; Collaborative Innovation Centre of Advanced Microstructures, Nanjing University, Nanjing 210093, China; School of Physical Science and Technology, ShanghaiTech University, Shanghai 201210, China; ShanghaiTech Laboratory for Topological Physics, Shanghai 200031, China; School of Emerging Technology and Department of Physics, University of Science and Technology of China, Hefei 230026, China; Department of Physics, University of Science and Technology of China, and Key Laboratory of Strongly Coupled Quantum Matter Physics, Chinese Academy of Sciences, Hefei 230026, China; Advanced Light Source, Lawrence Berkeley National Laboratory, Berkeley, CA 94720, USA; Korea Institute for Advanced Study, Seoul 02455, South Korea; Korea Institute for Advanced Study, Seoul 02455, South Korea; State Key Laboratory of Functional Materials for Informatics, Shanghai Institute of Microsystem and Information Technology (SIMIT), Chinese Academy of Sciences, Shanghai 200050, China; National Synchrotron Radiation Laboratory, University of Science and Technology of China, Hefei 230029, China; State Key Laboratory of Surface Physics, New Cornerstone Science Laboratory, and Department of Physics, Fudan University, Shanghai 200438, China; Shanghai Research Center for Quantum Sciences, Shanghai 201315, China; Institute for Nanoelectronic Devices and Quantum Computing, Fudan University, Shanghai 200433, China; Zhangjiang Fudan International Innovation Center, Fudan University, Shanghai 201210, China; State Key Laboratory of Surface Physics, New Cornerstone Science Laboratory, and Department of Physics, Fudan University, Shanghai 200438, China; Shanghai Qi Zhi Institute, Shanghai 200232, China; Shanghai Research Center for Quantum Sciences, Shanghai 201315, China; Institute for Nanoelectronic Devices and Quantum Computing, Fudan University, Shanghai 200433, China; Zhangjiang Fudan International Innovation Center, Fudan University, Shanghai 201210, China

**Keywords:** two-dimensional materials, 1T-TaSe_2_, dimensionality crossover, metal to Mott insulator transition

## Abstract

Two-dimensional materials represent a major frontier for research into exotic many-body quantum phenomena. In the extreme two-dimensional limit, electron-electron interaction often dominates over other electronic energy scales, leading to strongly correlated effects such as quantum spin liquid and unconventional superconductivity. The dominance is conventionally attributed to the lack of electron screening in the third dimension. Here, we discover an intriguing metal to Mott insulator transition in 1T-TaSe_2_ that defies conventional wisdom. Specifically, we find that dimensionality crossover, instead of reduced screening, drives the transition in atomically thin 1T-TaSe_2_. A dispersive band crossing the Fermi level is found to be responsible for the bulk metallicity in the material. Reducing the dimensionality, however, effectively quenches the kinetic energy of these initially itinerant electrons, and drives the material into a Mott insulating state. The dimensionality-driven metal to Mott insulator transition resolves the long-standing dichotomy between metallic bulk and insulating surface of 1T-TaSe_2_. Our work further reveals a new pathway for modulating two-dimensional materials that enables exploring strongly correlated systems across uncharted parameter space.

## Teaser

Researchers have revealed an unprecedent metallic state and dimensionality-driven metal to Mott-insulator transition in 1T-TaSe2, shedding light on a new pathway for modulating two-dimensional strongly-correlated materials.

## INTRODUCTION

Dimensionality plays a fundamental role in condensed matter physics. The reduction in dimensionality fundamentally alters the electronic structure of a material, often with profound consequences [1–3]. This is best exemplified by the myriad of novel phenomena discovered in two-dimensional (2D) materials, especially when the material family expands into major new branches of condensed matter research [4–6]. The emerging 2D strongly correlated materials bring new opportunities. Electron-electron interaction is generally enhanced in 2D [7,8]. This much-enhanced interaction, conventionally attributed to reduced screening in the 2D limit, enriches the already vast variety of novel electronic structures of 2D materials. Specifically, the many-body interaction (characterized by the on-site Coulomb energy *U*) offers a new tuning knob to the diverse single-particle physics in 2D materials that largely originates from the inter-site hopping of electrons (characterized by the width of the resulting energy band *W*). The competition/cooperation of the two energy scales may lead to unexpected quantum phenomena.

Correlated effects exist in various layered van der Waals crystals [5,9]. When thinned down to atomic thicknesses, these 2D crystals represent the thinnest possible materials that lend themselves to external modulations such as gate doping [10–12]. Recent discovery of moiré superlattices in van der Waals heterostructures has taken such tunability to unprecedented levels. For example, it has been demonstrated that both carrier doping and bandwidth *W* can be readily modulated by gate electric field [13–15]. Such tunability stems from the large moiré unit cell (consisting of thousands of atoms) that effectively reduces the electric field/doping required for modulating the correlated physics in the heterostructure [14,15]. The wide tunability comes, however, at a price—the large unit cell reduces both correlation energy scale *U* and bandwidth *W* to the order of ∼$10\, {{\rm meV}}$ [14–16]. Further exploring rich strongly correlated phenomena in 2D, as well as their potential applications, calls for fully-tunable material systems that retain a large energy scale.

Among correlated 2D materials, transition metal dichalcogenides 1T-TaX_2_ (X = S, Se) distinguish themselves as a potentially fully-tunable material system with a high correlation energy. The ground state of the materials features a peculiar star-of-David charge density wave (CDW) superlattice [17,18]. Each star-of-David unit cell contains 13 $5d$ conduction electrons contributed by the 13 Ta atoms in the cluster (Fig. 2b). The formation of the triangular superlattice, however, localizes all but one conduction electron on each cluster. The small star-of-David unit cell (compared to moiré unit cells in typical 2D heterostructures) dictates that the Coulomb energy of the lone electron remain high (up to ∼$500\,{{\rm meV}}$; ref [19,20]). Meanwhile, the unit cell is still large enough to ensure that the materials stay tunable—a charge doping of $7.3 \times {10^{13\,}}{\mathrm{c}}{{\mathrm{m}}^2}$ can fill/deplete the entire conduction band; this level of modulation is well within reach with ionic gating [21]. Indeed, signs of strong correlation have been observed in 1T-TaX_2_ and their heterostructures [19,20,22,23]. There are, however, still controversies on the nature of the insulating states in 1T-TaX_2_ [24–28], with the issues being particularly severe in 1T-TaSe_2_: although 1T-TaSe_2_ is metallic, its bulk and monolayer exhibit, at the Fermi level, a large spectral gap of a similar size [20,22,29]. The temperature-dependence of the gap resembled a thermally-driven Mott transition [30], but the underlying mechanism of the transition remains debated [31]. These problems represent a crucial missing piece to the strong correlation puzzle in 1T-TaX_2_. Their solution may provide important insights into 2D strongly correlated many-body physics in general.

Here, we directly address these open problems by probing the evolution of the electronic structure in 1T-TaSe_2_ as the dimensionality of the material is continuously lowered. At the three-dimensional (3D) bulk limit, we discover a dispersive band crossing the Fermi level, which has not been observed previously and thus solves the long running mystery of bulk metallicity in 1T-TaSe_2_. Meanwhile, at the 2D limit, we find unequivocal evidence that 1T-TaSe_2_ is, indeed, a Mott insulator. We further identify a metal to Mott insulator transition at a critical thickness of seven layers. Detailed analysis of angle-resolved photoemission spectroscopy (ARPES) and scanning tunneling spectroscopy (STS) data corroborated by first principles calculations reveals that the transition is driven by dimensionality crossover: lowering the dimensionality effectively reduces the width *W* of the dispersive band, and induces the transition when Coulomb energy *U* dominates over *W*. The dimensionality crossover is also at work on the surface of a bulk metal. The mechanism makes 1T-TaSe_2_ surface layers a Mott insulator, and resolves the dichotomy between metallic bulk and insulating surface of 1T-TaSe_2_. These results establish 1T-TaSe_2_ as a fully tunable, correlated 2D material with a high correlation energy.

## RESULTS AND DISCUSSION

**Figure 1.**
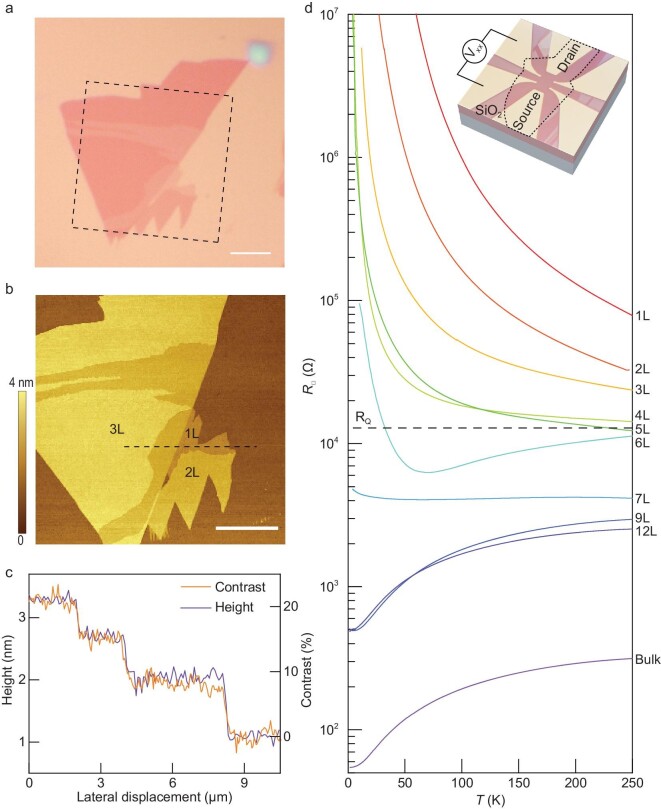
Metal to insulator transition in atomically thin 1T-TaSe_2_ flakes of varying thicknesses. (a) Optical image of a typical 1T-TaSe_2_ thin flake mechanically exfoliated on the substate. The substrate is Si wafer covered with 285-nm-thick SiO_2_. Scale bar, 5${\mathrm{\,\,\mu m}}$. (b) Atomic force microscopy (AFM) image of the area marked by the dashed square shown in (a). The number of layers, determined from AFM measurement of the sample thickness, is marked on various parts of the flakes (‘1L’, ‘2L’ and ‘3L’ denote monolayer, bilayer and trilayer, respectively). Scale bar, 5 μm. (c) Cross-sectional AFM height profile along the line cut marked by the black dashed line in (b). Superimposed on top is the optical contrast profile extracted from the optical image in (a) along the same line cut. The good agreement between the two profiles indicates that both methods—optical contrast and AFM—give accurate measurements of the sample thickness. (d) Temperature-dependent resistivity of 1T-TaSe_2_ flakes with varying number of layers. A metal to insulator transition is clearly visible at the critical thickness of seven layers. Data from bulk 1T-TaSe_2_ is also shown as a reference. The broken line denotes the value of quantum resistance ${R_Q} = h/2{e^2}$. Inset: Optical image of a bilayer 1T-TaSe_2_ device with schematic measurement setup sketched on top.

We start with the electronic transport characterization of 1T-TaSe_2_ as the sample thickness is varied from bulk down to monolayer. Thin flakes of 1T-TaSe_2_ are mechanically exfoliated on the Si substrates covered with 285 nm SiO_2_. The optical image of a representative few-layer 1T-TaSe_2_ flake is shown in Fig. 1a. We identify the number of layers from the optical contrast (Fig. 1a and c) in combination with an atomic force microscopy (AFM) measurement (Fig. 1b and c). Metal contacts are then defined on the flakes—by direct deposition of Cr and Au, typically 3 nm and 60 nm, respectively, through stencil masks—for subsequent transport measurements. Figure 1d displays the sheet resistance, *R*_□_, measured as a function of temperature, *T*, in 1T-TaSe_2_ flakes with varying thicknesses down to monolayer. The metallic (insulating) behavior of bulk (monolayer) 1T-TaSe_2_ is consistent with previous reports [20,29]. Remarkably, a metal-to-insulator transition (MIT) occurs at a critical thickness of seven layers. *R_□_* at the critical thickness is on the order of quantum resistance $h/2{e^2}$, and stays almost constant over the entire temperature range. The behavior signifies a quantum phase transition driven by dimensionality reduction. Here *h* is the plank constant and *e* the charge of an electron.

**Figure 2.**
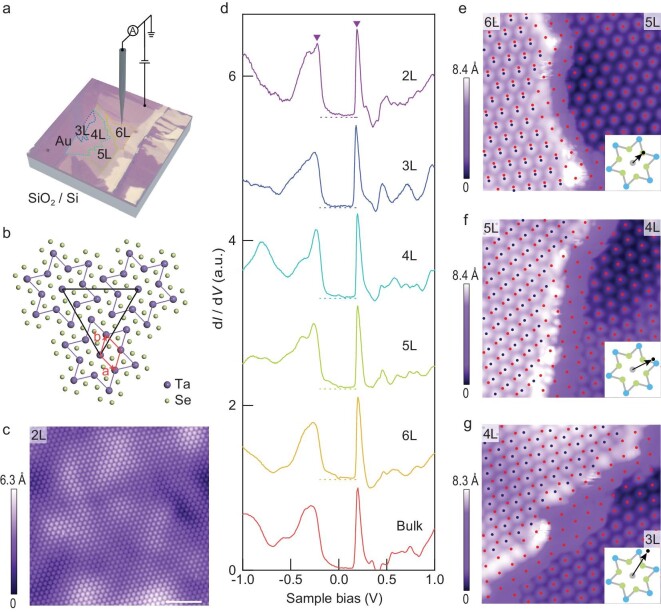
Scanning tunnelling microscopy and spectroscopy of few-layer 1T-TaSe_2_ in the star-of-David CDW phase. (a) Few-layer 1T-TaSe_2_ flake exfoliated on Au-covered SiO_2_ substrate probed by STM (STM tip is sketched). Number of layers determined from optical contrast and STM measurements is marked on different parts of the flake. (b) Top view of the monolayer 1T-TaSe_2_ crystal structure. Every 13 Ta atoms form a star-of-David cluster (outlined in purple). The CDW superlattice is formed by the star-of-David clusters arranged in a triangular lattice. (a and b) denote the in-plane unit vectors of the atomic lattice. (c) Constant-current STM topography of a bilayer 1T-TaSe_2_ (${V_{\mathrm{b}}} = 0.3\,\,{\mathrm{V}}$, ${I_{\mathrm{t}}} = 50\,\,{\mathrm{pA}}$). The large-scale ripples reflect corrugations of the substrate. Scale bar, 10 nm. (d) Differential conductance spectra acquired on 1T-TaSe_2_ flakes with varying number of layers (${V_{\mathrm{b}}} = 1\,\,{\mathrm{V}}$, ${I_{\mathrm{t}}} = \,\,200\,\,{\mathrm{pA}}$, ${V_{{\mathrm{r}}.{\mathrm{m}}.{\mathrm{s}}.}} = 5\,\,{\mathrm{mV}}$). Spectra are vertically displaced for clarity. Broken lines mark the zero of each curve. All spectra show a spectral gap (bounded by the two peaks marked by the two triangles on each curve), which does not vary with sample thickness. (e–g) Constant-current STM topography (${V_{\mathrm{b}}} = \,\,0.3\,\,{\mathrm{V}}$, ${I_{\mathrm{t}}} = \,\,50\,\,{\mathrm{pA}}$) of a 6 L to 5 L step edge, a 5 L to 4 L step edge, and a 4 L to 3 L step edge. Centers of the star-of-David clusters are marked by blue (red) dot array on the upper (lower) terraces in each panel. By extrapolating the position of the array on the lower terrace to the upper terraces, we extract the stacking order between the two terraces, which is depicted in the inset of each panel. Insets: Schematic illustration of the stacking order determined from STM topographies. The black dot and arrow indicate the lattice shift of the upper terrace relative to the lower terrace. All STM and tunneling spectroscopy data were taken at $T = 4.3{\mathrm{\,\,K}}$.

The dimensionality-driven MIT observed in transport, however, does not translate to a spectroscopic transition in scanning tunneling microscopy and spectroscopy (STM/STS) measurements. Figure 2d displays the differential conductance (${\mathrm{d}}I/{\mathrm{d}}V$) spectra, which are proportional to energy-resolved local density of states (LDOS), on 1T-TaSe_2_ flakes with varying number of layers. The spectra were obtained at $T\,\, = \,\,4.3{\mathrm{\,\,K}}$ on atomically-clean surfaces, as exemplified in Fig. 2c, with an experimental setup sketched in Fig. 2a. Surprisingly, the bulk crystal and all few-layer specimens exhibit a spectral gap of the same size at the Fermi level ${E_F}$, bracketed by two pronounced peaks that have been referred to as the Hubbard bands [19,22,32]; no clear transition was visible in the spectra of few-layer samples. (The monolayer spectrum differs from those of thicker samples, probably due to coupling with the conductive Au substrate; Supplementary Fig. 5b). We note that the few-layer spectra seem to be independent of CDW stacking order. Different stacking orders yield identical spectra in few-layer samples (Fig. 2e–g).

**Figure 3.**
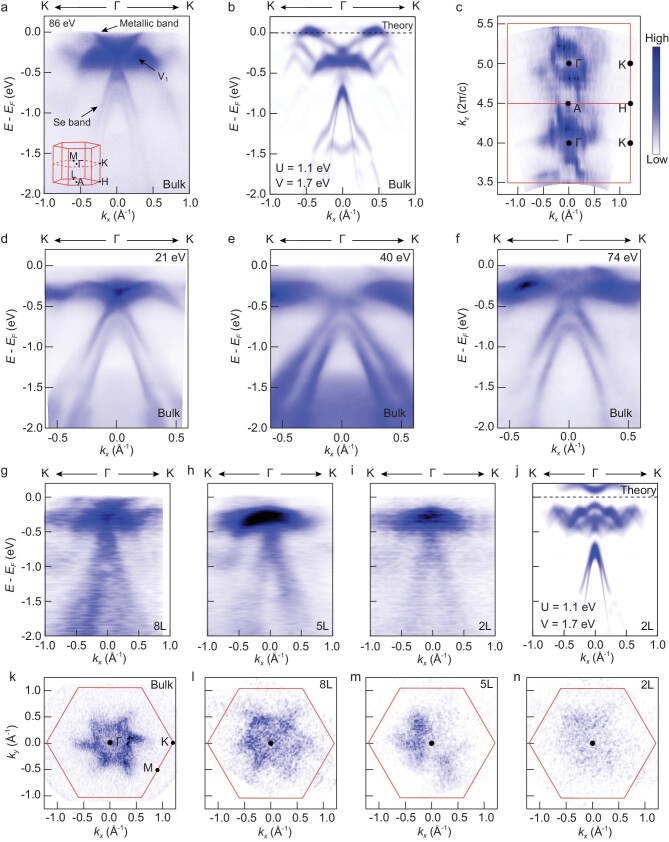
Electronic structure of bulk and few-layer 1T-TaSe_2_ probed by ARPES. (a) ARPES spectrum of bulk 1T-TaSe_2_ acquired with 86 eV *p*-polarized photons at $T = 20{\,\,{\rm K}}$ along Γ–K direction (at ${k_z} = \,\,0$) of the undistorted (i.e. no CDW) atomic lattice Brillouin zone. The full Brillouin zone is sketched in the inset. Apart from the previously reported V1 band and Se band (in, e.g. ref [48]), we discover an additional dispersive band that crosses the Fermi level at ${k_x}\ 0.3\,{{\rm AA}^{ - 1}}$. (b) DFT + *U* + *V* band structure of bulk 1T-TaSe_2_. The calculation is performed on the star-of-David CDW superlattice with $\ 2{\bf{a}} + {\bf{c}}$ interlayer stacking order. The calculated band structure was unfolded onto the undistorted atomic lattice Brillouin zone for comparison with ARPES spectrum from (a). The calculation reproduces all main features of the ARPES spectrum, including the dispersive metallic band. (c) Spectral weight mapping near the Fermi level ([$- 10{\mathrm{\,\,meV}}$, $10{\mathrm{\,\,meV}}$]) in the ${k_x} - {k_z}$ plane of bulk 1T-TaSe_2_ taken at $T\,\, = {\mathrm{\,\,}}20{\mathrm{\,\,K}}$. Here ${k_x}$ is defined along the Γ–K direction. A Fermi surface having a period of $2\pi /c$ in ${k_z}$ is clearly visible. (d–f) ARPES spectra along the Γ–K direction of undistorted atomic lattice Brillouin zone of bulk 1T-TaSe_2_ acquired at photon energies of $21\,\,{\mathrm{eV}}$ (d) $40\,\,{\mathrm{eV}}$ (e) and $74\,\,{\mathrm{eV}}$ (f). The three photon energies correspond to out-of-plane wavevectors of $2.69 \times ( {2\pi /c} )$,${\mathrm{\,\,}}3.69 \times ( {2\pi /c} )$ and $4.69 \times ( {2\pi /c} )$, respectively. (g–i) ARPES spectra along the Γ–K direction (at ${k_z} = \,\,0$) of undistorted atomic lattice Brillouin zone of 8-layer (8 L; g) 5-layer (5 L; h) and bilayer (2 L; i) 1T-TaSe_2_ flakes. Data were taken with $86\,\,{\mathrm{eV}}$ circularly-polarized photons at $T\,\, = {\mathrm{\,\,}}40{\mathrm{\,\,K}}$ in a nano-ARPES setup. (j) DFT + *U* + *V* band structure of bilayer 1T-TaSe_2_ with $2{\bf{a}} + {\bf{c}}$ CDW stacking order for comparison with the ARPES spectrum from (i). (k–n) Spectral weight mapping acquired with $86{\mathrm{\,\,eV}}$ photons near the Fermi level ([$- 1{\mathrm{\,\,meV}}$, $1{\mathrm{\,\,meV}}$]) in the ${k_x} - {k_y}$ plane of bulk (h), 8 L (i), 5 L (j) and 2 L (k) 1T-TaSe_2_.

The seemingly contradictive results from transport and STS measurements prompt us to examine in detail the electronic structure of bulk 1T-TaSe_2_ with ARPES. Figure 3a displays the ARPES spectrum along the Γ–K direction acquired with $86{\mathrm{\,\,eV}}$  *p*-polarized photons at $T\,\, = \,\,20{\mathrm{\,\,K}}$. The spectrum reproduces the main features of bulk electronic structure reported previously: Se $4p$ band, CDW band folding, and the flat feature (referred to as ‘V1’ band) centered around $- 300\,\,{\mathrm{meV}}$, which encompasses the lower Hubbard band [30,33]. Surprisingly, however, we observe an additional, dispersive, band that crosses the Fermi level, forming a well-defined Fermi surface centered at the Γ point (Fig. 3k). This metallic band is 3D in nature, i.e. the band also disperses strongly in ${k_z}$, in stark contrast to the 2D Se $4p$ band (Supplementary Fig. 8). In particular, the band switches from electron-like (Fig. 3a) to hole-like, and back to electron-like (Supplementary Fig. 7a and b), as ${k_z}$ traverses the entire Brillouin zone from Γ to A, and back to Γ. The strong ${k_z}$ dispersion is corroborated by a Fermi surface that is periodic in ${k_z}$ (Fig. 3c); an observed periodicity of $2\pi /c$ (*c* is the out-of-plane lattice constant) further indicates the absence of superstructures, such as dimerization [24–26], in the out-of-plane direction. Finally, we note that the metallic band is visible only in ARPES spectra taken at high photon energies. Figure 3d–f displays the ARPES spectra along the Γ–K direction acquired with incident photon energies of $21\,\,{\mathrm{eV}}$, $40\,\,{\mathrm{eV}}$ and $74\,\,{\mathrm{eV}}$ on a same bulk 1T-TaSe_2_ crystal. The metallic band is absent at the photon energy of 21 eV (Fig. 3d), but is present at higher photon energies (Fig. 3e and f). Such photon energy dependence, which is attributable to cross section and the matrix element effects [34,35], may be the reason why the metallic band eluded previous ARPES detection [30,33,36].

The metallic band resolves the mystery of metallicity in bulk 1T-TaSe_2_. But the contradiction between the metallic band and the gapped tunneling spectrum on the same bulk sample remains. A consistent picture emerges once we consider the fact that ARPES has a penetration depth of approximately $1{\mathrm{\,\,nm}}$ (∼2 layers; ref [37]), whereas the STM signal is dominated by the surface layer. The ARPES is, therefore, able to access 1T-TaSe_2_ bulk, in addition to the surface layer which is probed by both ARPES and STM. Our results indicate that 1T-TaSe_2_ is a bulk metal covered with an insulating surface.

These findings raise a fundamental question: what is the nature of the insulating state? Our experimental evidence indicates that strong correlation is responsible for generating the spectral gap at the Fermi level. Key insights come from nano-ARPES measurements of atomically thin 1T-TaSe_2_ flakes (Supplementary Fig. 9). Figure 3g–i displays the ARPES spectra along the Γ–K direction of 1T-TaSe_2_ flakes of varying thickness down to bilayer (see Methods for details of the measurements). The spectra again reproduce familiar features of the bulk, i.e. Se $4p$ band, CDW band folding and the V1 band, all of which do not vary appreciably with sample thickness. The newly discovered metallic band, however, gradually disappears, giving way to an energy gap bounded by the V1 band, as the sample becomes thinner (Fig. 3g–i). The same transition is reflected in the vanishing Fermi surfaces of the flakes (Fig. 3k–n); the absence of the metallic band around the A point in a trilayer indicates that the Fermi surface is gapped globally in the 2D limit (Supplementary Fig. 10). The vanishing metallic band corroborates the MIT that we observed in transport measurements of few-layer 1T-TaSe_2_. More importantly, the way that the band disappears (and the spectral gap develops) reveals important clues on the nature of the spectral gap. There are two main points to notice. First, no energy shift is detected in either the dispersive metallic band or the V1 band in the thin flake samples. This observation rules out band bending effect [38] as a cause of the spectral gap.

Second, specimens with even and odd number of layers all exhibit the same spectral gap (Figs. 2d, 3h and 3i). Meanwhile, a constant spectral gap, as opposed to alternating large and small gaps, was observed in the tunneling spectroscopy performed on 1T-TaSe_2_ terraces with various layer configurations (Fig. 2e–g). These observations, combined with an absence of out-of-plane superstructures in bulk 1T-TaSe_2_ flakes, rules out layer dimerization [24] as the origin of the gap in 1T-TaSe_2_. Other possibilities of a single-particle gap, such as quantum confinement and weak localization [39,40], are similarly excluded. In fact, we find that no known single-particle mechanism consistently explains the observed insulating state on the surface of metallic 1T-TaSe_2_ bulk. On the other hand, once electron correlation is considered, the measured spectral gap is naturally explained by a Mott gap. Because 1T-TaSe_2_ has a cluster CDW superlattice that is exactly half-filled, initially itinerant electrons at the material surface (or in few-layers) will make the transition to a Mott insulating state, once the onsite Coulomb energy dominates over the kinetic energy of the electrons.

The question next arises as to what drives the metal to Mott insulator transition both at bulk surface and in few-layer 1T-TaSe_2_. Prior to answering this question, we first point out that all main features of the ARPES spectra are captured by our density functional theory calculations with self-consistently obtained onsite Hubbard *U* and inter-site Hubbard *V* interactions [41,42] (DFT + *U* + *V*; see Methods). Specifically, calculations of the total energy indicate that the energetically most favorable stacking orders are $\pm 2{\bf{a}} + {\bf{c}}$, which yield identical DFT + *U* + *V* band structures (Supplementary Table 1). The DFT + *U* + *V* calculations reproduce the dispersive metallic band in the bulk and the gapped spectrum in the bilayer, which agree well with experimental observations (Fig. 3b and j). The calculations further reveal that both the dispersive band and the lower Hubbard band derive from the ${d_{{z^2}}}$ orbital of the central Ta atom in each star-of-David cluster (Supplementary Fig. 12). Even though the intra-layer hopping of the ${d_{{z^2}}}$ electrons in the CDW phases are strongly suppressed because of the relatively large lateral dimension of the star-of-David cluster, strong inter-layer hopping remains between the pancake-shaped clusters. The inter-layer hopping gives rise to a half-filled dispersive band that hosts itinerant electrons, whose Mott localization turns the material into a Mott insulator.

**Figure 4.**
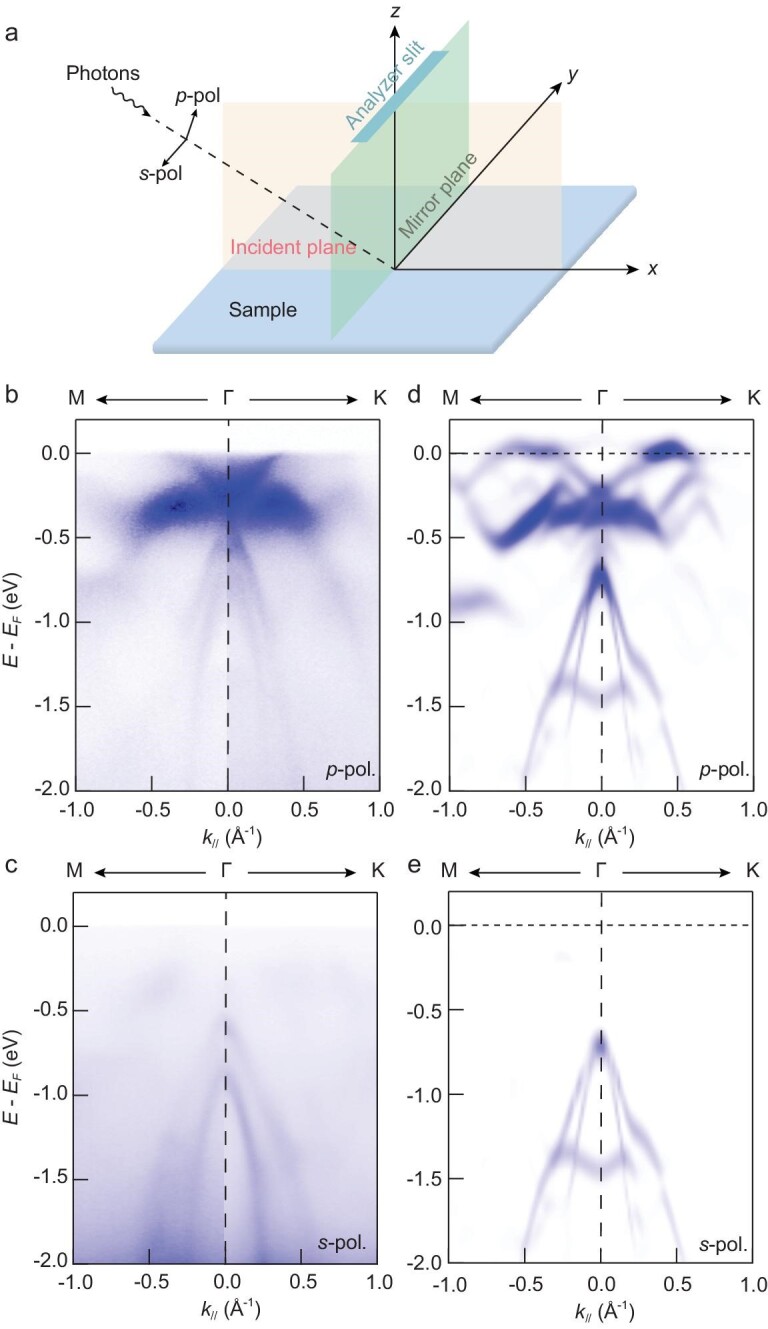
Orbital character of electronic bands in bulk 1T-TaSe_2_ probed by polarized ARPES. (a) Schematic illustration of our polarized ARPES measurement setup. The analyzer slit is perpendicular to the incident plane (orange). The mirror plane (green) is defined by the analyzer slit direction and the sample surface normal. (b and c), ARPES spectra along the Γ–K and Γ-M (at ${k_z} = \,\,0$) of bulk 1T-TaSe_2_ acquired with $86{\mathrm{\,\,eV}}$ *p* (b) and *s* (c) polarized light at $T\,\, = {\mathrm{\,\,}}20{\mathrm{\,\,K}}$. (d and e) DFT + *U* + *V* band structure of bulk 1T-TaSe_2_ for comparison with polarized ARPES spectra from (b and c), respectively. Panels (d and e) depict the calculated band structure with and without Ta ${d_{{z^2}}}$ orbital contribution, respectively.

This revelation is strongly supported by polarized ARPES measurements performed on the surface of 1T-TaSe_2_ bulk. Figure 4a illustrates our experimental setup of polarized ARPES, where photons impinge on the sample surface with either *p* (in the incident plane) or *s* (perpendicular to the incident plane) polarization. Symmetry dictates that electronic states with odd (even) parity with respect to the mirror plane can be observed with *p* (*s*) polarized photons [35,43]. In addition, the *p*-polarized photons have an out-of-plane (*z*) component, which leads to a large ARPES cross section for states with pronounced out-of-plane character such as the Ta ${d_{{z^2}}}$ orbital. The *s-*polarized photons, on the other hand, lie completely in the *x*–*y* plane, and are therefore more sensitive to orbitals with in-plane characters [44,45]. We observe that both the V1 band and the dispersive metallic band are visible with *p*-polarized photons, but are absent with *s*-polarized photons (Fig. 4b and d). This observation confirms our calculation that the two bands originate from Ta ${d_{{z^2}}}$ orbitals.

We are now poised to explain the underlying mechanism that drives the metal to Mott insulator transition. Physics at the Mott transition is essentially governed by the relative strength of kinetic energy of electrons (characterized by *W*) over onsite Coulomb energy *U*, i.e. $W/U$. Here $U\,\,$represents the bare Coulomb energy cost when an additional electron hops into a star-of-David cluster in 1T-TaSe_2_. The system is expected to turn into a Mott insulator when $W/U$ falls below a critical value [46–48]. We first rule out the hypothesis that the reduced dielectric screening in few-layer 1T-TaSe_2_ enhances *U*, and drives the Mott transition. Because the size of the spectral gap seen in STS and ARPES provides a direct measure of Coulomb energy in the Mott insulating state, we are able to extract *U* as a function of sample thickness (Figs. 2d, 3d–f and Supplementary Fig. 14). We discover that *U* remains nearly constant as the sample approaches bilayer thickness; the same spectral gap is observed on 1T-TaSe_2_ bulk surface. We have also calculated, self-consistently, the onsite Coulomb energy of the center Ta *d*-orbitals, $\tilde U$, with varying number of layers (Supplementary Fig. 14). The thickness-independent $\tilde U$ obtained from the calculations supports our experimental observations, and further rules out Coulomb energy as a driving force of the Mott transition.

**Figure 5.**
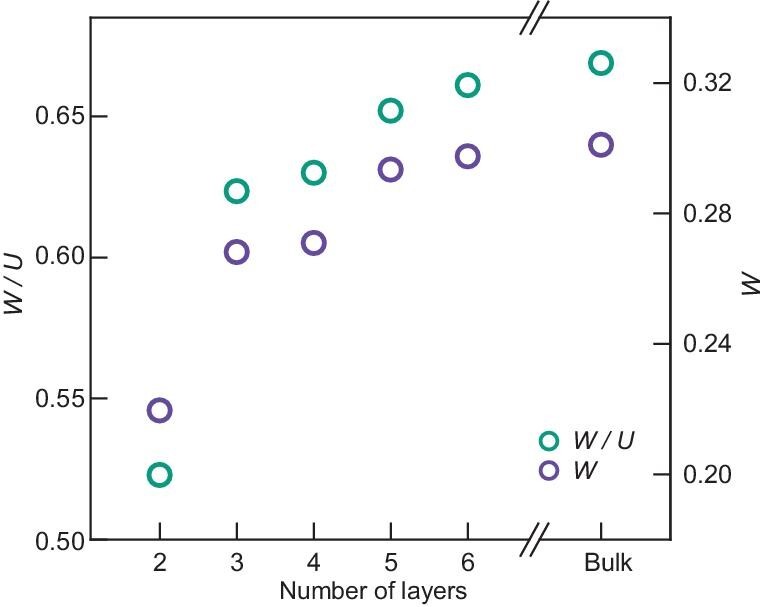
Narrowing of the metallic band and metal to Mott insulator transition in few-layer 1T-TaSe_2_. The bandwidth of the metallic band, *W*, and the ratio between *W* and the onsite Coulomb energy,${\mathrm{\,\,}}W/U$, are plotted as functions of the number of layers. Here *W* is obtained from DFT + *U* + *V* non-magnetic calculations of the metallic band, and *U* is extracted from the size of the spectral gap seen in STS. The $W/U$ in few-layer 1T-TaSe_2_ fall within the range where metal to Mott insulator transition is anticipated (see text).

Our first-principles calculations, however, reveal a significantly suppressed bandwidth *W* in atomically thin 1T-TaSe_2_. Figure 5 displays the width of the dispersive metallic band calculated in a nonmagnetic configuration before electron correlation is added. The bulk band width of ∼$300{\mathrm{\,\,meV}}$ gradually narrows down to ∼$220{\mathrm{\,\,meV}}$ as a result of reduced average inter-layer coordination number for the star-of-David clusters, as the sample thickness approaches bilayer. Consequently, the ratio $W/U$ monotonically increases from 0.52 (in bilayer) to 0.67 (in bulk), a range that coincides with theoretically predicted $W/U$ range (0.56 to 0.75) where metal to Mott insulator transition takes place (Fig. 5; ref [46–48]). It now becomes clear that band narrowing under reduced dimensionality drives the Mott transition in 1T-TaSe_2_. The dimensionality-driven Mott transition also naturally explains the Mott insulating surface layer on bulk 1T-TaSe_2_: reduced average coordination number at the bulk surface causes a similar dimensionality crossover, inducing a 2D Mott insulating state at the surface. The dimensionality-driven Mott transition resembles the transition observed near room temperature [30], but the underlying mechanisms are completely different—here the Mott transition takes place at low temperatures without the temperature-induced modulation of the atomic lattices. Finally, we note that even though we only considered coordination number reduction, slight lattice distortions such as swelling in *c*-axis (seen in 1T-TaS_2_ thin flakes [49]) may potentially play a role in suppressing the bandwidth in few-layer samples. Such distortions are beyond the detection limit in the present study, and future work is needed to delineate their contribution to the Mott transition.

## CONCLUSION

To summarize, we discover an intriguing metal to Mott insulator transition that is driven by dimensionality reduction. Specifically, we provide unequivocal evidence that the insulating phase in few-layer 1T-TaSe_2_ and on bulk 1T-TaSe_2_ surface is a strongly correlated Mott insulator, which evolves from a 3D metallic band that we observe for the first time in bulk 1T-TaSe_2_. Below a critical thickness of seven layers, reduced average coordination number effectively suppresses the bandwidth (i.e. the kinetic energy of the electron) while the large correlation energy remains almost intact, which turns the initially bulk metal into a 2D correlated Mott insulator. A similar dimensionality crossover induces a Mott insulating layer on the bulk surface, resolving the long-standing dichotomy between the metallic bulk and insulating surface in 1T-TaSe_2_. These results establish few-layer 1T-TaSe_2_ as a fully tunable material system with relatively high correlation energy. Our work further sheds new light on alternative mechanisms of modulating correlated 2D materials, where various emergent correlated quantum phenomena such as quantum spin liquid and high-temperature superconductivity may be investigated in the extreme 2D limit.

## METHODS

### Growth and characterization of bulk 1T-TaSe_2_ crystal

High-quality 1T-TaSe_2_ single crystals were grown by the chemical vapor transport (CVT) method (see Supplementary Note 1 for growth details). The as-grown 1T-TaSe_2_ single crystals typically have a dimension of $4{\mathrm{\,\,mm\,\,}} \times {\mathrm{\,\,}}4{\mathrm{\,\,mm\,\,}} \times {\mathrm{\,\,}}0.2{\mathrm{\,\,mm}}$ (Supplementary Fig. 1a). Supplementary Fig. 1b and 1d display the X-ray diffraction pattern and Raman spectroscopy of a typical crystal, respectively. The results indicate the good crystal quality of bulk 1T-TaSe_2_.

### STM and STS measurements on 1T-TaSe_2_ thin flakes

We used oxidized Si wafers covered with 2 nm of Cr and 3 nm of Au as substrates, on which we prepared atomically clean thin 1T-TaSe_2_ flakes for STM measurements in two steps. We first pressed pieces of bulk 1T-TaSe_2_ crystal, fixed on a piece of vacuum-compatible tape, onto the substrate in a glovebox. The water and oxygen level in the glovebox was kept below 0.1 ppm during the process. The substrate, along with the 1T-TaSe_2_ crystal and the tape, was subsequently transferred into ultra-high vacuum (UHV). We then peeled away the tape under pressures below $5 \times {10^{ - 9}}$ mbar, leaving atomically clean few-layer 1T-TaSe_2_ on the substrate (see Supplementary Note 2 for details of sample thickness determination). Our STM and STS measurements were performed in a Createc low-temperature STM at $T\,\, = {\mathrm{\,\,}}4.3{\mathrm{\,\,K}}$ in UHV environment with base pressure blow${\mathrm{\,\,}}2 \times {10^{ - 10}}$ mbar. We used electrochemically etched polycrystalline tungsten tips in all our STM measurements. All tips were calibrated by tunnelling differential conductance (${\mathrm{d}}I/{\mathrm{d}}V$) measurements on Au (111) surface before STM measurements on 1T-TaSe_2_. ${\mathrm{d}}I/{\mathrm{d}}V$ spectra were obtained through lock-in detection with an excitation wiggle voltage ${V_{{\mathrm{r}}.{\mathrm{m}}.{\mathrm{s}}.}}$ ranging from $5{\mathrm{\,\,to\,\,}}10{\mathrm{\,\,mV}}$ at frequency $f\,\, = \,\,444\,\,{\mathrm{Hz}}$.

### ARPES measurements

ARPES measurements on freshly cleaved bulk 1T-TaSe_2_ crystals were performed at 03 U and 09 U beamlines of Shanghai Synchrotron Radiation Facility (SSRF; the polarized ARPES was performed at 09 U beamline in particular). Nano-ARPES measurements on 1T-TaSe_2_ thin flakes were conducted at 07 U beamline of SSRF (see Supplementary Note 8 for more details). All end stations are equipped with Scienta Omicron DA30 electron analyzers, which have an angular resolution better than $0.2^\circ $. The energy resolution is $30{\mathrm{\,\,meV}}$ at 03 U and 09 U, and $35{\mathrm{\,\,meV}}$ at 07 U. All ARPES data were acquired at temperatures below the CDW transition temperature of $473{\mathrm{\,\,K}}$ ($20{\mathrm{\,\,K}}$ at 03 U and 09 U, and $40{\mathrm{\,\,K}}$ at 07 U) under ultrahigh vacuums better than $8.0 \times {10^{ - 11}}\,\,{\mathrm{Torr}}$. Some of the preliminary ARPES data was collected at the BL 10.0.1 of the Advanced Light Source.

### First-principles calculations of the electronic structure

First-principles density-functional theory calculations (referred to as DFT + *U* + *V*) were performed with Quantum Espresso package and Garrity-Bennett-Rabe-Vanderbilt ultrasoft pseudopotentials [50]. The kinetic energy cutoff for charge density was set to 200 Ry, and $5 \times 5 \times 5$ k-point mesh was used for self-consistent calculations of $\sqrt {13} $ by $\sqrt {13} $ by two star-of-David CDW structures for all stacking orders under investigation. The CDW structures were fully relaxed with the rev-vdW-DF2 functional [51] (see Supplementary Note 10 for more details).

## Supplementary Material

nwad144_Supplemental_File
