# Supplementary material for: Dimensionality-driven metal to Mott insulator transition in two-dimensional 1T-TaSe2
Source: Natl Sci Rev. 2023 May 16;11(3):nwad144. doi: 10.1093/nsr/nwad144 (PMC11640825; doi:10.1093/nsr/nwad144)
Supplement: nwad144_Supplemental_File [file nwad144_supplemental_file.docx]

Supplementary Information for

**Dimensionality-driven Metal to Mott Insulator Transition in Two-dimensional 1T-TaSe_2_**

Ning Tian, Zhe Huang, Bo Gyu Jang, Shuaifei Guo, Ya-Jun Yan, Jingjing Gao, Yijun Yu, Jinwoong Hwang, Cenyao Tang, Meixiao Wang, Xuan Luo, Yuping Sun, Zhongkai Liu, Dong-Lai Feng, Xianhui Chen, Sung-Kwan Mo, Minjae Kim, Young-Woo Son^*^, Dawei Shen^*^, Wei Ruan^*^ and Yuanbo Zhang^*^

^*^Correspondence should be addressed to Y.Z. ([zhyb@fudan.edu.cn](mailto:zhyb@fudan.edu.cn)), W.R. ([weiruan@fudan.edu.cn](mailto:weiruan@fudan.edu.cn)), D.S. ([dwshen@ustc.edu.cn](mailto:dwshen@ustc.edu.cn)) and Y.-W.S. ([hand@kias.re.kr](mailto:hand@kias.re.kr))

**Content**

1. **Growth and characterization of bulk 1T-TaSe_2_ crystal**
2. **Determining the flake thickness in STM measurements**
3. **Orbital texture of the Mott insulating state in few-layer 1T-TaSe_2_**
4. **STM/STS of monolayer 1T-TaSe_2_ mechanically exfoliated on Au substrate**
5. **The hole-like metallic band in** **the** $\boldsymbol{k}_{\boldsymbol{x}}\boldsymbol{-}\boldsymbol{k}_{\boldsymbol{y}}$ **plane at** $\boldsymbol{k}_{\boldsymbol{z}}\boldsymbol{\sim\pi/c}$ **of bulk 1T-TaSe_2_**
6. **Variations of the dispersive metallic band in the** $\boldsymbol{k}_{\boldsymbol{z}}$ **direction**
7. **Two-dimensional nature of the Se** $\boldsymbol{4}\boldsymbol{p}$ **bands**
8. **Nano-ARPES intensity maps of the few-layer 1T-TaSe_2_ flakes**
9. **The absence of the hole-like metallic band at** $\boldsymbol{k}_{\boldsymbol{z}}\boldsymbol{\sim\pi/c}$ **in trilayer 1T-TaSe_2_**
10. **First-principles calculations of the electronic structure**
11. **The energetically most favorable stacking order in bulk 1T-TaSe_2_**
12. **DFT+*U*+*V* band structure of bulk 1T-TaSe_2_ along the A-H direction**
13. **Orbital character of the V1 band and metallic band in DFT+*U*+*V* calculations**
14. **DFT+*U*+*V* calculation of the width of the metallic band in the nonmagnetic configuration**
15. **Values of** $\boldsymbol{U}$ **and** $\boldsymbol{W}$ **in bulk and few-layer 1T-TaSe_2_**
16. **References**
17. **Growth and characterization of bulk 1T-TaSe_2_ crystal**

High-purity elements were stoichiometrically mixed and sealed under vacuum ($3\times{10}^{-5}$ mbar) in a quartz ampoule with a very small quantity of iodine as the transport agent (7‰ mass fraction). The ampoules were then placed horizontally into a tube furnace. The hot and cold ends of the tube were kept at 1050 ℃ and 950 ℃, respectively, during growth. To achieve high crystal quality and to minimize defect density, the ampoules were kept at growth temperature for one month. The ampoules were then rapidly quenched in cold saturated salt-water (~ -15 ℃) at the end of the growth.


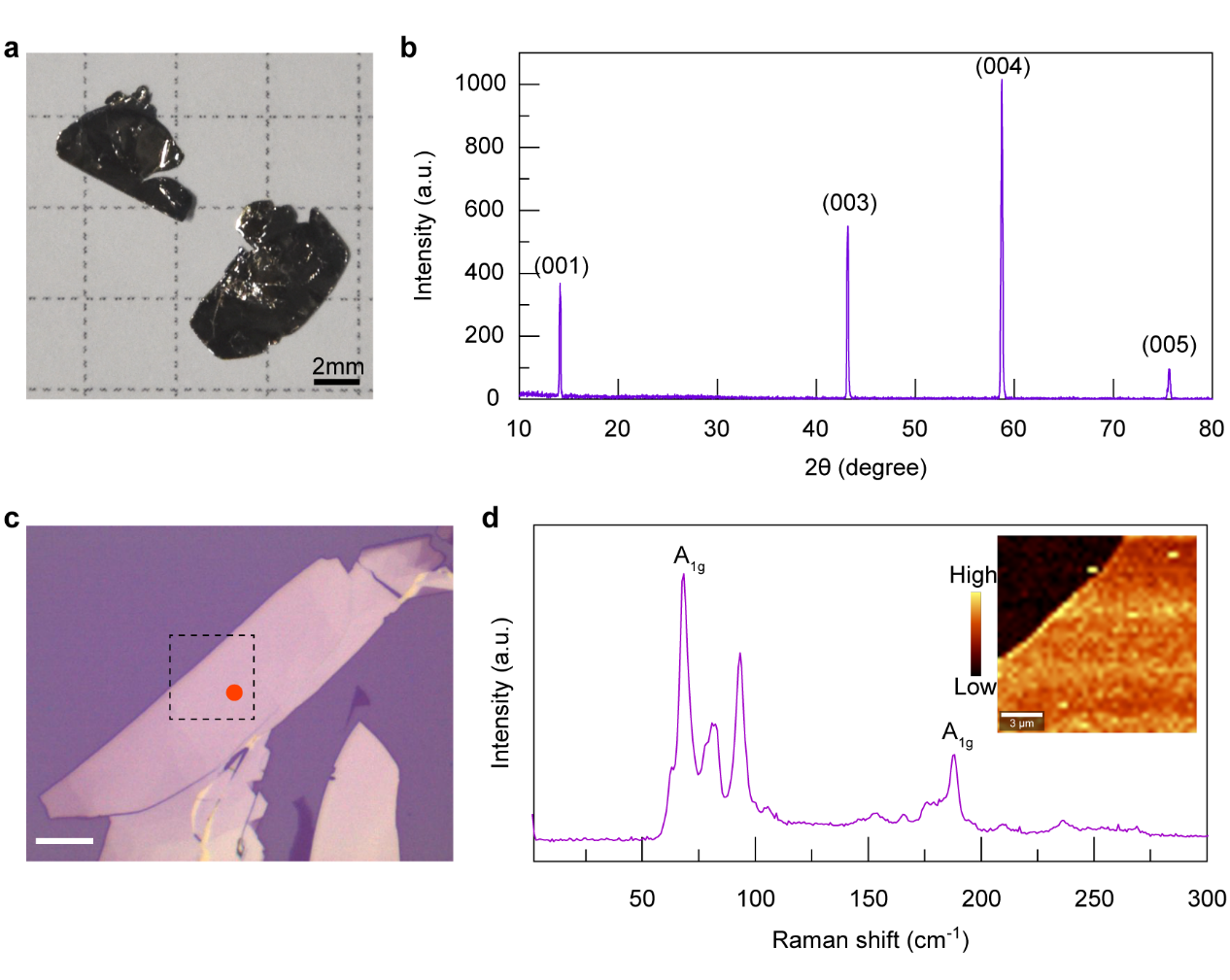


**Supplementary Fig. 1│Characterization of bulk 1T-TaSe_2_. a**, Optical image of typical bulk 1T-TaSe_2_ crystals grown by CVT method (see Methods). **b**, X-ray diffraction pattern of bulk 1T-TaSe_2_ single crystals. Data were obtained by Bruker D8 A25 Discover diffractometer with Cu Kα radiation. **c**, Optical image of a typical piece of thick 1T-TaSe_2_ crystal mechanically exfoliated onto the substate. The substrate is a Si wafer covered with 285-nm-thick SiO_2_. Scale bar, 10 $\mu m$. **d**, Raman spectroscopy of the crystal at the position marked by the red dot in **a**. Inset: 2D Raman mapping of the 1T-TaSe_2_ Raman active A_1g_ mode at 188 $\mathrm{cm}^{-1}$. The mapping area is marked by the broken square in **c**.


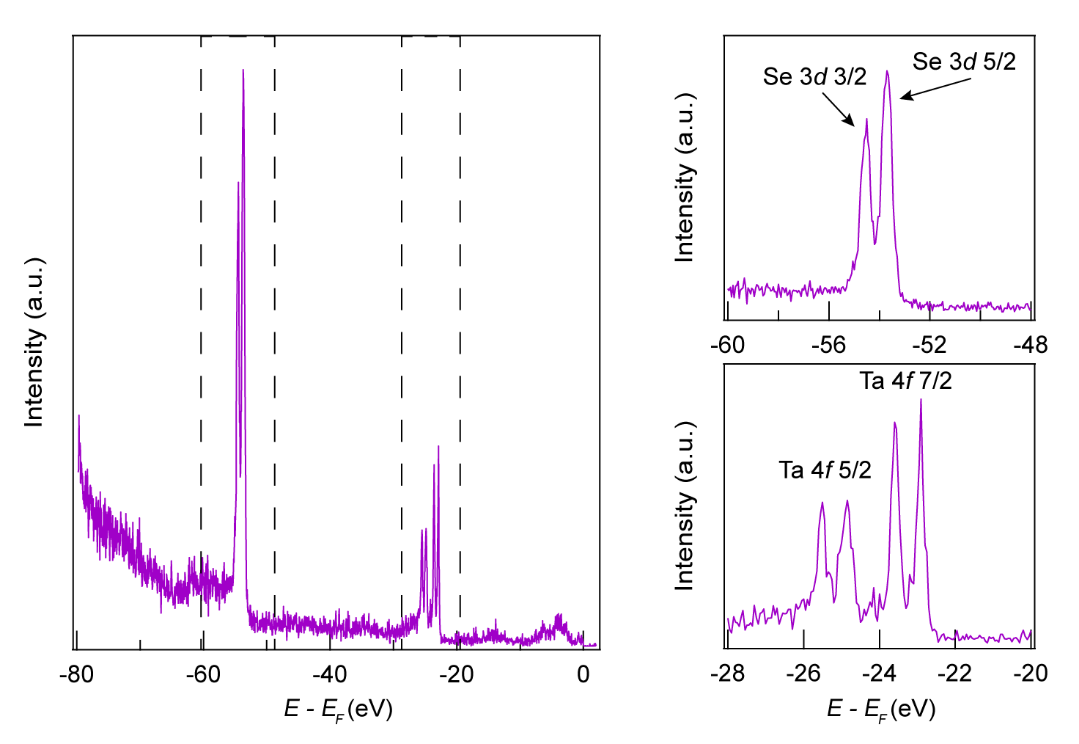


**Supplementary Fig. 2│X-ray photoelectron spectroscopy of bulk 1T-TaSe_2_.** The spectrum was acquired at 20 K with a photon energy of 104 eV. Only Ta and Se peaks are observed within the experimental resolution.

1. **Determining the flake thickness in STM measurements**

Because the transport and STM/STS experiments require different sample preparations, we used slightly different procedures to fabricate and characterize samples for the two experiments. Specifically, the thickness of samples used for transport measurement was determined by optical contrast and AFM, while the thickness of samples used for STM/STS measurement, which required atomically clean surface, was characterized by optical contrast (as shown in Supplementary Fig. 3, an image taken under ultra-high vacuum) and in situ STM imaging to prevent sample surface degradation. We determined the sample thickness by tracing the tip height change at each step edge (~ 600 nm; Fig. 2e-g) during continuous lateral tip movement on the sample surface, and by combining this data the optical contrast results. The thickness of the samples ranged from 3 to 6 layers as shown in Fig. 2.


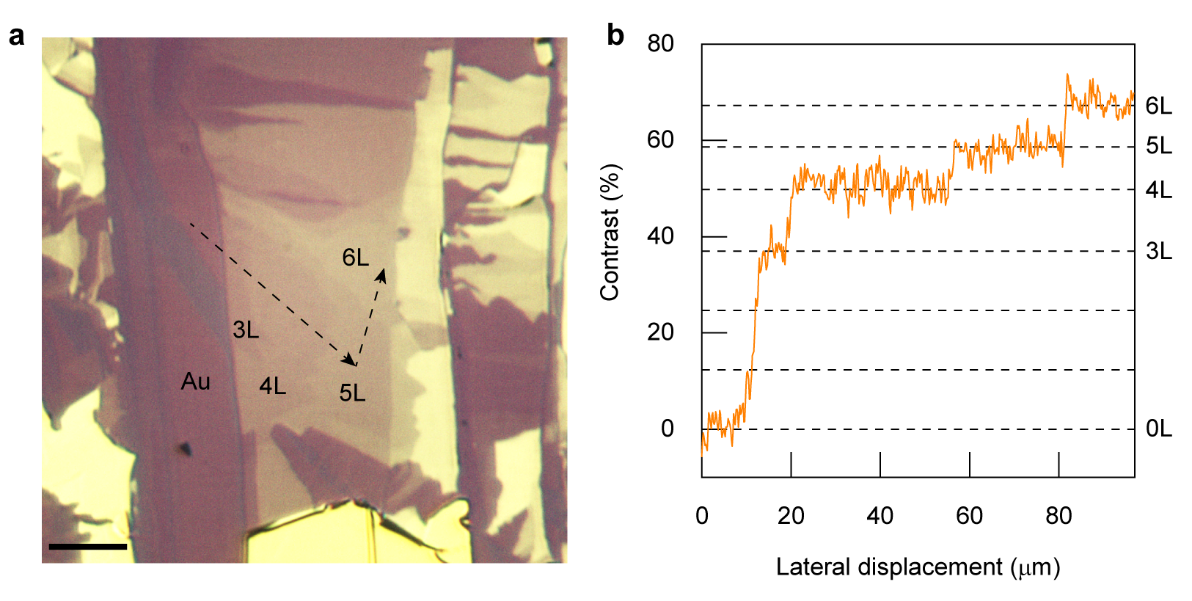


**Supplementary Fig. 3│Determining the thickness of the STM sample. a**, Optical image of a typical 1T-TaSe_2_ thin flake used for STM measurements. The substrate is Au-covered SiO_2_. Scale bar, 20$\mu m$. **b**, Cross-sectional optical contrast profile along the line cut marked by the black dashed line in **a**. The optical contrast profile, combined with step edges identified by STM scanning along the line cut, yields an accurate determination of the sample thickness.

1. **Orbital texture of the Mott insulating state in few-layer 1T-TaSe_2_**

We probe the orbital texture of few-layer 1T-TaSe_2_ by performing $dI/dV$ spatial mapping of its energy-dependent orbital texture. The $dI/dV$ maps of two pronounced peaks at around $V_{b}=\pm0.2 V$, which have been referred to as the lower and upper Hubbard bands^1^, show enhanced local density of states (LDOS) at the center of each star-of-David (Supplement Fig. 2b). This observation is consistent with the interpretation of two peaks as the two Hubbard bands in a Mott insulator [1, 2].


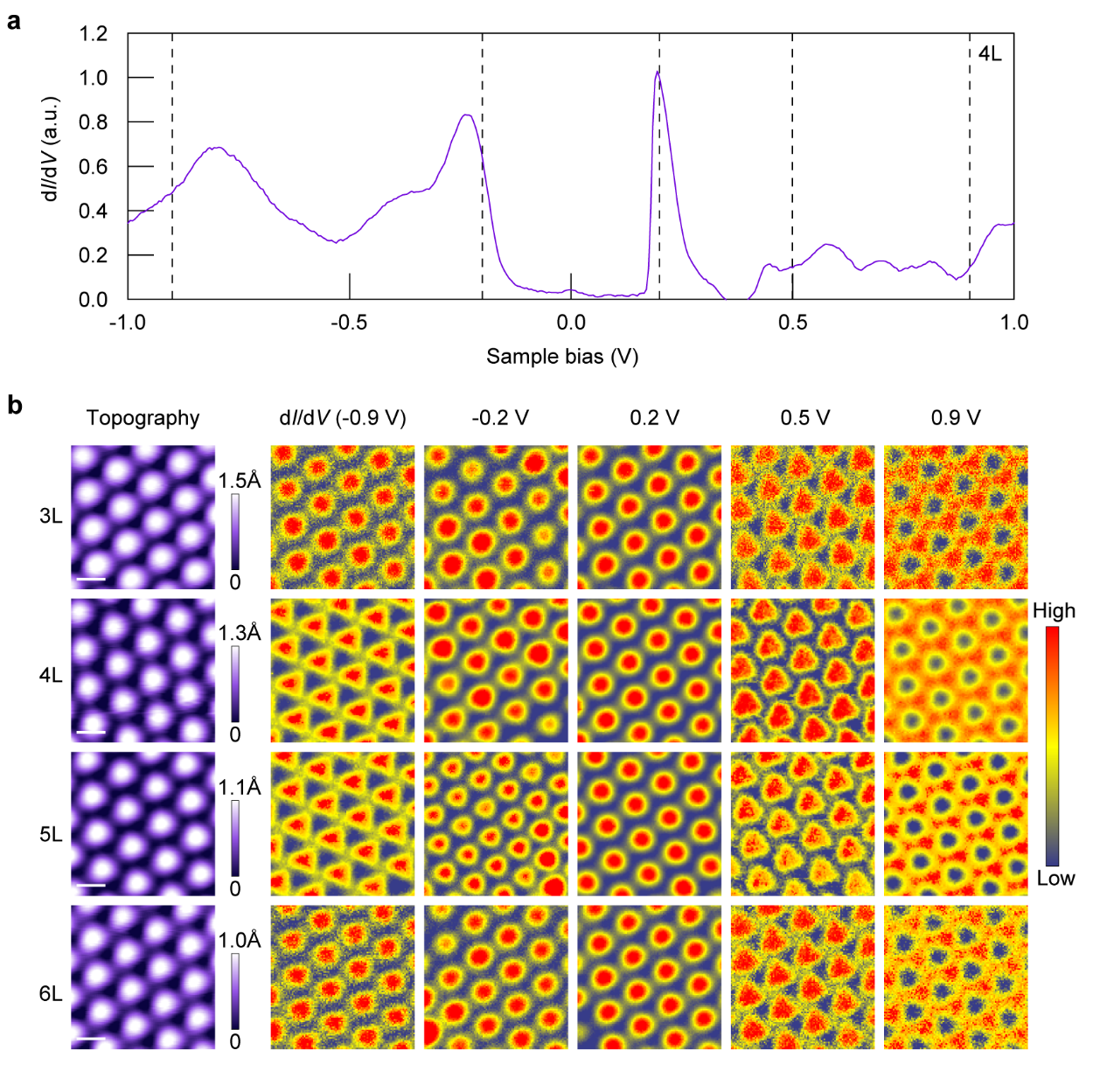


**Supplementary Fig. 4│Energy-resolved orbital texture of few-layer 1T-TaSe_2_ probed by STM/STS. a**, The differential conductance $dI/dV$ spectrum of four-layer 1T-TaSe_2_ ($V_{b}=1V$, $I_{t}=200 pA$, $V_{r.m.s.}=5\mathrm{mV}$). **b**, STM topographs ($V_{b}=0.5 V$, $I_{t}=100 \mathrm{pA}$) of few-layer 1T-TaSe_2_ with varying number of layers (leftmost column) and $dI/dV$ conductance maps acquired on the same areas at various bias voltages ($V_{b}=1V$, $I_{t}= 100 pA$). $V_{r.m.s.}=5 mV$in 3L and 6L measurements, and $V_{r.m.s.}=8 mV$in 4L and 5L measurements. Scale bar, 1nm. Data were obtained at $T=4.3 K$. Triangular superlattices of the star-of-David clusters are clearly visible on every topographs. The $dI/dV$ maps at $V_{b}=\pm0.2 V$ indicate that the LDOS at the energies of the lower and upper Hubbard bands are localized at the centers of the star-of-David clusters.

1. **STM/STS of monolayer 1T-TaSe_2_ mechanically exfoliated on Au substrate**


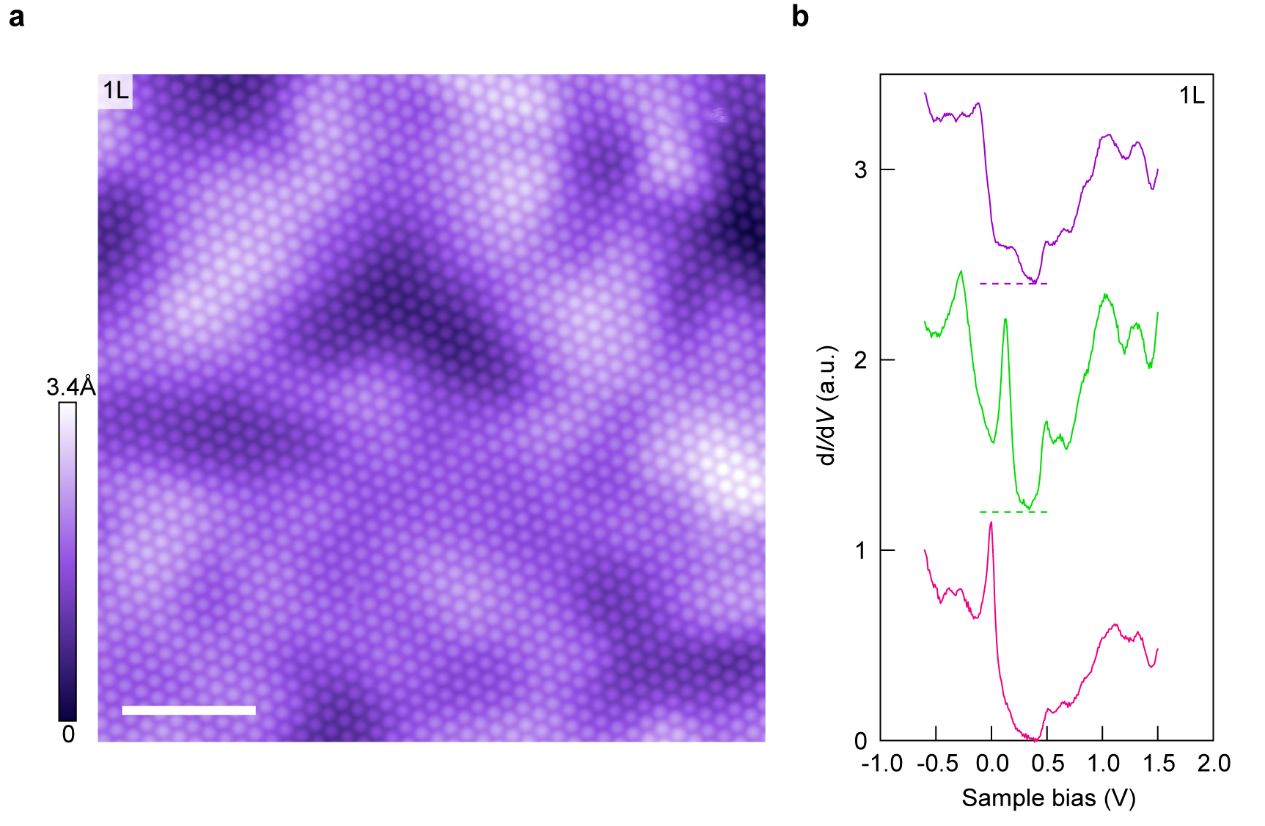


**Supplementary Fig. 5│STM topograph and differential conductance (**$\mathbf{d}\boldsymbol{I}\mathbf{/d}\boldsymbol{V}$**) spectra of monolayer 1T-TaSe_2_ flake. a**, STM topograph of a typical monolayer 1T-TaSe_2_ flake ($V_{b}=0.5 V$, $I_{t}=200 \mathrm{pA}$). The flake was mechanically exfoliated on a SiO_2_/Si wafer. The wafer was coated with 2 nm of Cr and 3 nm of Au through thermal evaporation prior to the exfoliation. The monolayer flake exhibits an atomically clean surface with the triangular star-of-David CDW superlattice clearly visible. The large-scale corrugations on the sample surface reflect the corrugations of the Au substrate. Scale bar, $10 nm$. **b**, Typical $dI/dV$ spectra ($V_{b}=-0.6 V$, $I_{t}=200 \mathrm{pA}$, $V_{r.m.s.}=10 \mathrm{mV}$) acquired at different locations on monolayer 1T-TaSe_2_ flake. The spectra, vertically offset for clarity, exhibit large variations that stem from the spatial inhomogeneity in the electronic structure of monolayer 1T-TaSe_2_ on amorphous Au film. The spectra differ from those of the few-layer 1T-TaSe_2_ shown in Fig. 2. They also differ from that of the monolayer 1T-TaSe_2_ grown on epitaxial bilayer-graphene terminated 6H-SiC (0001) with molecular beam epitaxy [3]. We attribute the differences to the coupling between the 1T-TaSe_2_ and the disordered Au substrate, which may cause strong disturbances to the electronic structure of the monolayer. All data were obtained at $T=4.3 K$. In a bilayer 1T-TaSe_2_, finite tunneling probability between the tip and the bottom metallic 1T-TaSe_2_ layer, which strongly couples to the Au substrate, gives rise to an enhanced low energy DOS. This finite tunneling exponentially decays with increasing sample thickness, resulting in the decreasing low-energy DOS from 2L to 3L.

1. **The hole-like metallic band in** **the** $\boldsymbol{k}_{\boldsymbol{x}}\boldsymbol{-}\boldsymbol{k}_{\boldsymbol{y}}$ **plane at** $\boldsymbol{k}_{\boldsymbol{z}}\boldsymbol{\sim\pi/c}$ **of bulk 1T-TaSe_2_**


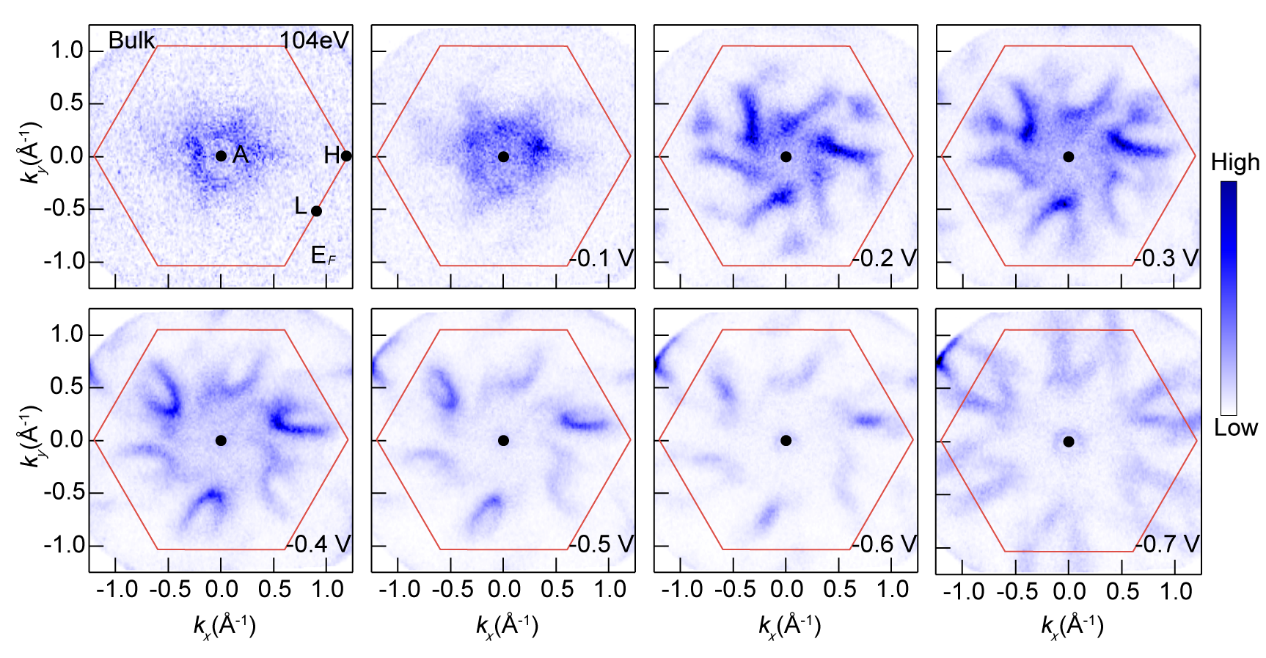


**Supplementary Fig. 6│Spectral weight mapping of the metallic band in** **the** $\boldsymbol{k}_{\boldsymbol{x}}\boldsymbol{-}\boldsymbol{k}_{\boldsymbol{y}}$ **plane at** $\boldsymbol{k}_{\boldsymbol{z}}\boldsymbol{\sim\pi/c}$ **of bulk 1T-TaSe_2_ at various energies.** Data were acquired with $104 eV$ *p*-polarized photons, and the spectral weight maps were obtained in [$-25 meV$, $25 meV$] intervals at energies indicated on each panel. All data were taken at $T=20 K$.

1. **Variations of the dispersive metallic band in the** $\boldsymbol{k}_{\boldsymbol{z}}$ **direction**


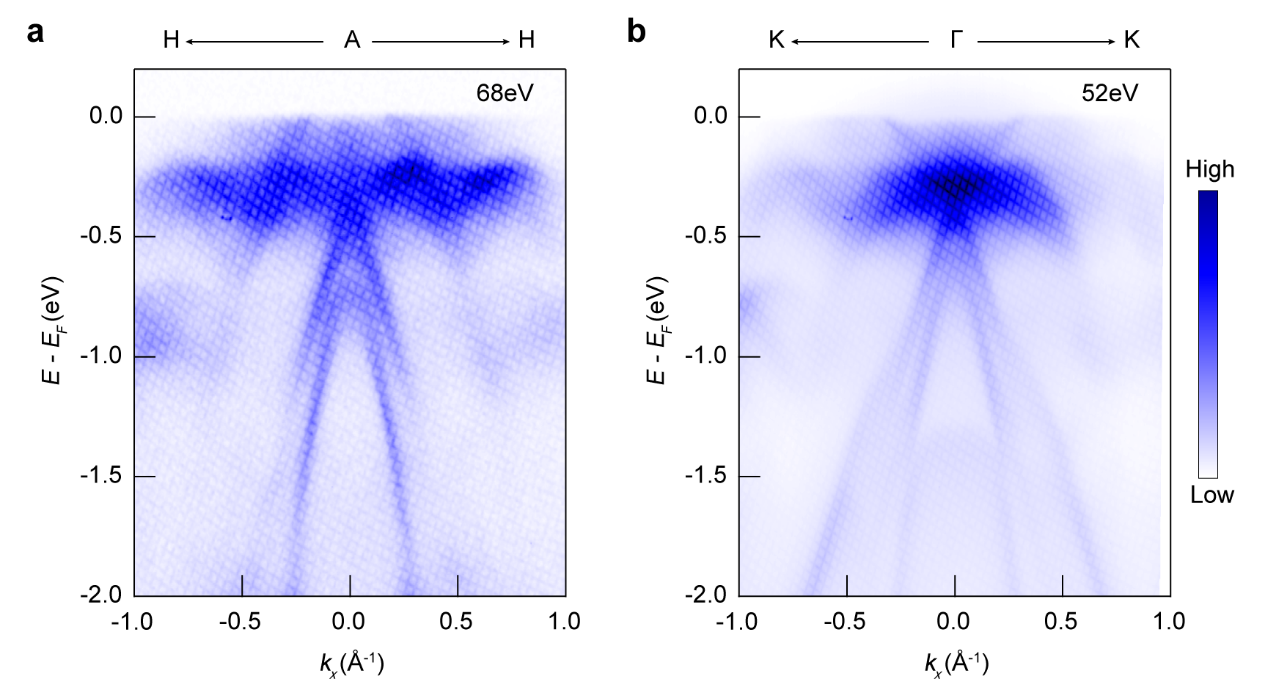


**Supplementary Fig. 7│ARPES spectra along A-H (at** $\boldsymbol{k}_{\boldsymbol{z}}\boldsymbol{=\pi/c}$**) and Γ-K (****at** $\boldsymbol{k}_{\boldsymbol{z}}\boldsymbol{=}\mathbf{0}$**) of the undistorted atomic lattice Brillouin zone of bulk 1T-TaSe_2_. a**, ARPES spectrum along the A-H direction probed by $68 eV$ photons. The metallic band becomes hole-like at $k_{z}=\pi/c$. **b**, ARPES spectrum along the Γ-K direction probed by $52 eV$ photons. The in-plane dispersion of the metallic band switches from electron-like (at $k_{z}=0$; Fig. 3a) to hole-like (at $k_{z}=\pi/c$; panel **a**), and back to electron-like (at $k_{z}=0$; panel **b**) as $k_{z}$ traverses the entire Brillouin zone. The similarity between the spectra at $h\nu=86 eV$ and $h\nu=52 eV$ reflects the periodicity of $2\pi/c$ in the bulk’s electronic structure along $k_{z}$. All data were taken at $T=20 K$.

1. **Two-dimensional nature of the Se** $\boldsymbol{4}\boldsymbol{p}$ **bands**


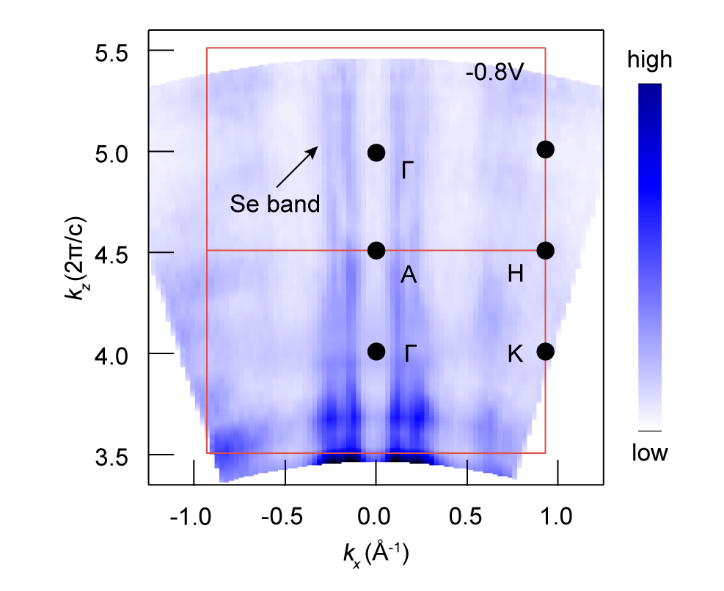


**Supplementary Fig. 8│Dispersion of the Se** $\boldsymbol{4}\boldsymbol{p}$ **bands along** $\boldsymbol{k}_{\boldsymbol{z}}$ **in bulk 1T-TaSe_2_.** Spectral weight mapping in the $k_{x}-k_{z}$ plane of bulk 1T-TaSe_2_ at an energy of $E=-0.8 eV$ below the Fermi level. The spectral weight was obtained in a $20 meV$ energy window around $E$. The Se $4p$ bands manifest as straight lines along Γ-A direction. The lack of $k_{z}$ dependence reflects the 2D nature of the Se $4p$ bands.

1. **Nano-ARPES intensity maps of the few-layer 1T-TaSe_2_ flakes**

The Nano-ARPES has a spatial resolution of $400 nm$, which enables us to probe the local ARPES intensity within a sample (the same type of thin flake sample used in STM measurements; typical size $\sim50 \mu m\times50 \mu m$). Locating the thin flakes in the nano-ARPES setup, however, poses a challenge. To this end, we marked the position of the sample area with a sharp tip before loading the samples into the measurement setup. We then scanned the sample area and acquired a map of the ARPES intensity at a photoelectron energy of $86 eV$. The large ARPES intensity from Au substrate gives a clear contrast between the sample and the substrate. The intensity map resolves the profile of the flakes, which allows us to perform ARPES measurements on areas with uniform thickness.


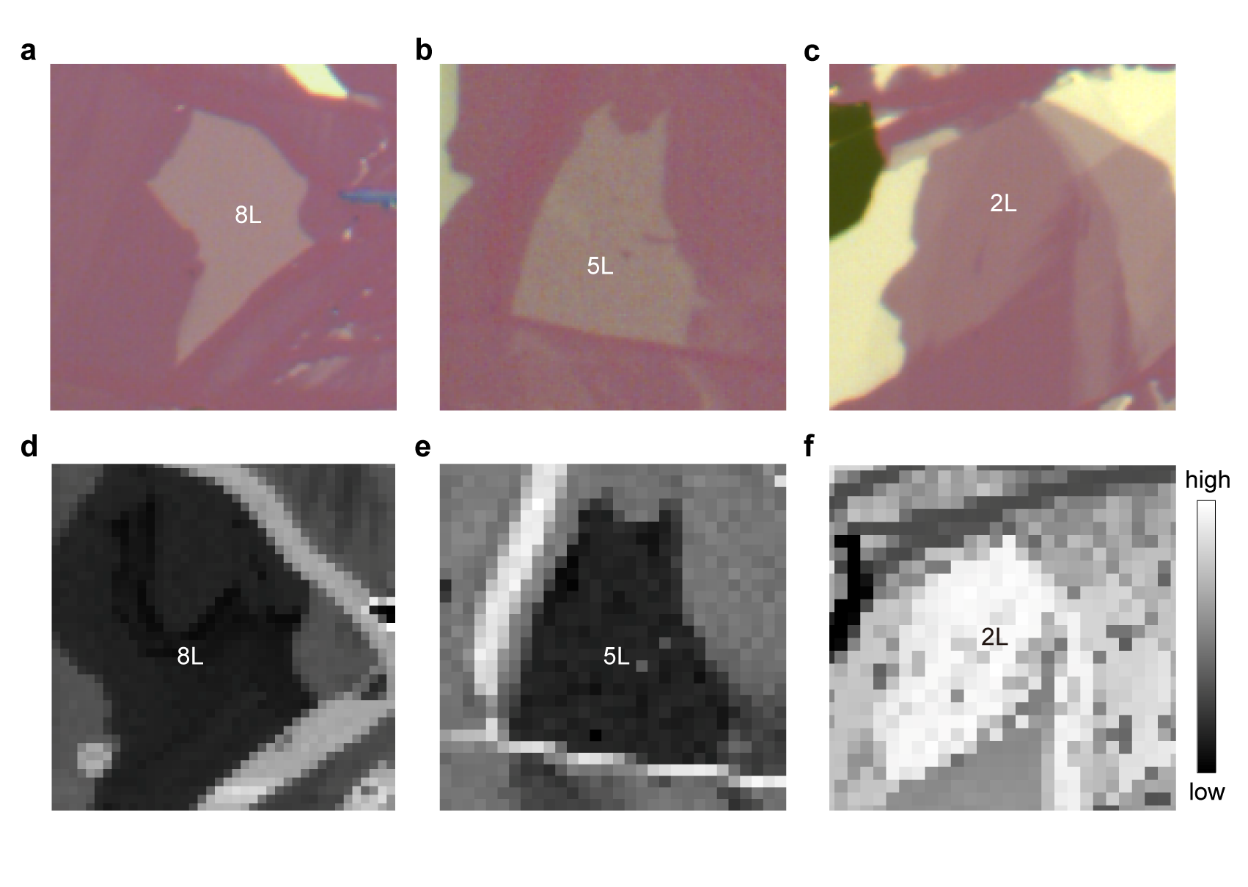


**Supplementary Fig.** **9│****Optical image and nano-ARPES intensity map of few-layer 1T-TaSe_2_ flakes**. **a-c**, Optical images of 8L, 5L and 2L 1T-TaSe_2_ flakes. **d** and **e**, maps of nano-ARPES intensity (integrated electronic structure below Fermi level [$-2 eV$, $0\mathrm{eV}$]) on the same region shown in **a** and **b**. **f**, nano-ARPES intensity map of integrated electronic structure obtained from the Se core level on the same region shown in **c**.

1. **The absence of the hole-like metallic band at** $\boldsymbol{k}_{\boldsymbol{z}}\boldsymbol{\sim\pi/c}$ **in trilayer 1T-TaSe_2_**


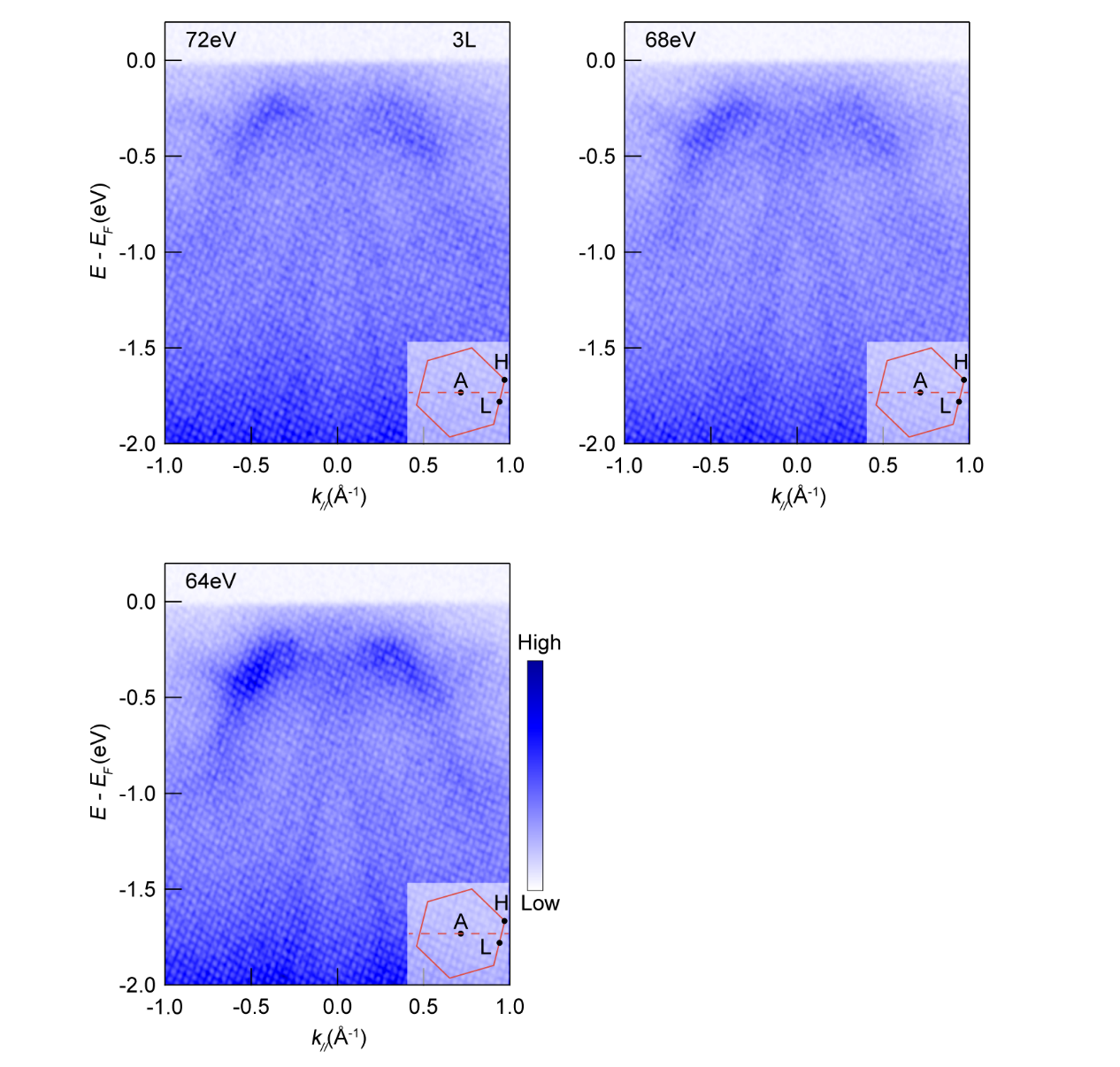


**Supplementary Fig. 10│ARPES spectra of trilayer 1T-TaSe_2_ at** $\boldsymbol{k}_{\boldsymbol{z}}\boldsymbol{= \pi/c}$**.** ARPES spectra show the in-plane band structure of trilayer 1T-TaSe_2_ along the dashed line of the undistorted atomic lattice Brillouin zone. All three spectra acquired at photon energies of $72 eV$, $68 eV$ and $64 eV$ probe the band structure at $k_{z}= \pi/c$. The hole-like metallic band disappears, giving rise to a spectral gap around $A$ point in the trilayer sample.

1. **First-principles calculations of the electronic structure**

We adopted the newly developed pseudo-hybrid density functionals for self-consistently obtained extended Hubbard interaction to calculate the electronic structure [4, 5]. This method has been shown to describe the quasiparticle energy bands of various solids such as semimetal, semiconductor, ionic insulator and Mott insulator with an accuracy comparable to the *GW* approximation [6]. The self-consistently computed Hubbard parameters are consistent with those obtained with other methods [6, 7]. The method also describes the structural properties for semiconducting silicon, diamond and germanium crystals as well as Mott insulating NiO and MnO very well [7, 8]. In particular, the computed phonon dispersion for Mott insulators NiO and MnO agree well with experimental results and dynamical mean field theory calculations [8]. These examples have demonstrated the capability of in calculating the structural property and the electronic structure, as well as the coupling between the two, in correlated systems such as 1T-TaSe_2_.

The onsite Hubbard $\tilde{U}$ and inter-site Hubbard $V$ parameters are self-consistently obtained from undistorted $1\times1$ bulk structure. The obtained $\tilde{U}$ value for Ta $d$ orbital is $1.1 eV$, and the $V$ value between Ta $d$ and Se $p$ orbitals vary from 1.7 to $2.2 eV$ depending on their bond direction for a bulk case. For direct comparison with ARPES spectra, the band structure of the CDW superlattice were unfolded into the Brillouin zone of undistorted $1\times1$ structure. The unfolding was performed using the BandUP code [9, 10]. The band broadening was simulated with an imaginary part of self-energy of the form $a\omega^{2}+b$, where $a=1.0 {eV}^{-1}$, $b=0.1 eV$, and 𝜔 is an energy difference between the Fermi energy and band energy.

1. **The energetically most favorable stacking order in bulk 1T-TaSe_2_**


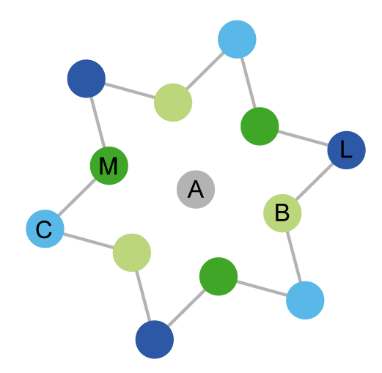


| Stacking order | | meV/star-of-David |
| --- | --- | --- |
| $\mathbf{c}$ | **A** | 43.78 |
| $\mathbf{a}\boldsymbol{+}\mathbf{c}$ | **B** | 33.38 |
| $2\mathbf{a}\boldsymbol{+}\mathbf{c}$ | **C** | -0.10 |
| $\boldsymbol{-}\mathbf{a}\boldsymbol{+}\mathbf{c}$ | **M** | 33.26 |
| $\boldsymbol{-}2\mathbf{a}\boldsymbol{+}\mathbf{c}$ | **L** | 0.00 |

**Supplementary Table 1│Total energy calculated for all five possible stacking orders in bulk** **1T-TaSe_2_.** The energies were computed relative to the value of the $-2\mathbf{a}+\mathbf{c}$ stacking order, which is set to be 0 (see Methods for details of the calculations). Here the in-plane lattice vector $\mathbf{a}$ is shown in Fig. 2b, and $\boldsymbol{c}$ denotes the lattice vector in the out-of-plane direction. The five stacking orders are also referred to as **A**, **B**, **C**, **M** and **L** in the literature [11], which are illustrated in the right panel. Our first-principles calculations show that the $2\mathbf{a}+\mathbf{c}$ and $-2\mathbf{a}+\mathbf{c}$ stacking orders have a similar total energy, and the two stacking orders have the lowest total energies compared among all five possible stacking configurations. DFT+*U*+*V* calculations yield identical electronic structures for $\pm2\mathbf{a}+\mathbf{c}$ stacking orders. The DFT+*U*+*V* band structures of $2\mathbf{a}+\mathbf{c}$ stacked bulk and few-layer 1T-TaSe_2_ are shown throughout this manuscript.

1. **DFT+*U*+*V* band structure of bulk 1T-TaSe_2_ along the A-H direction**


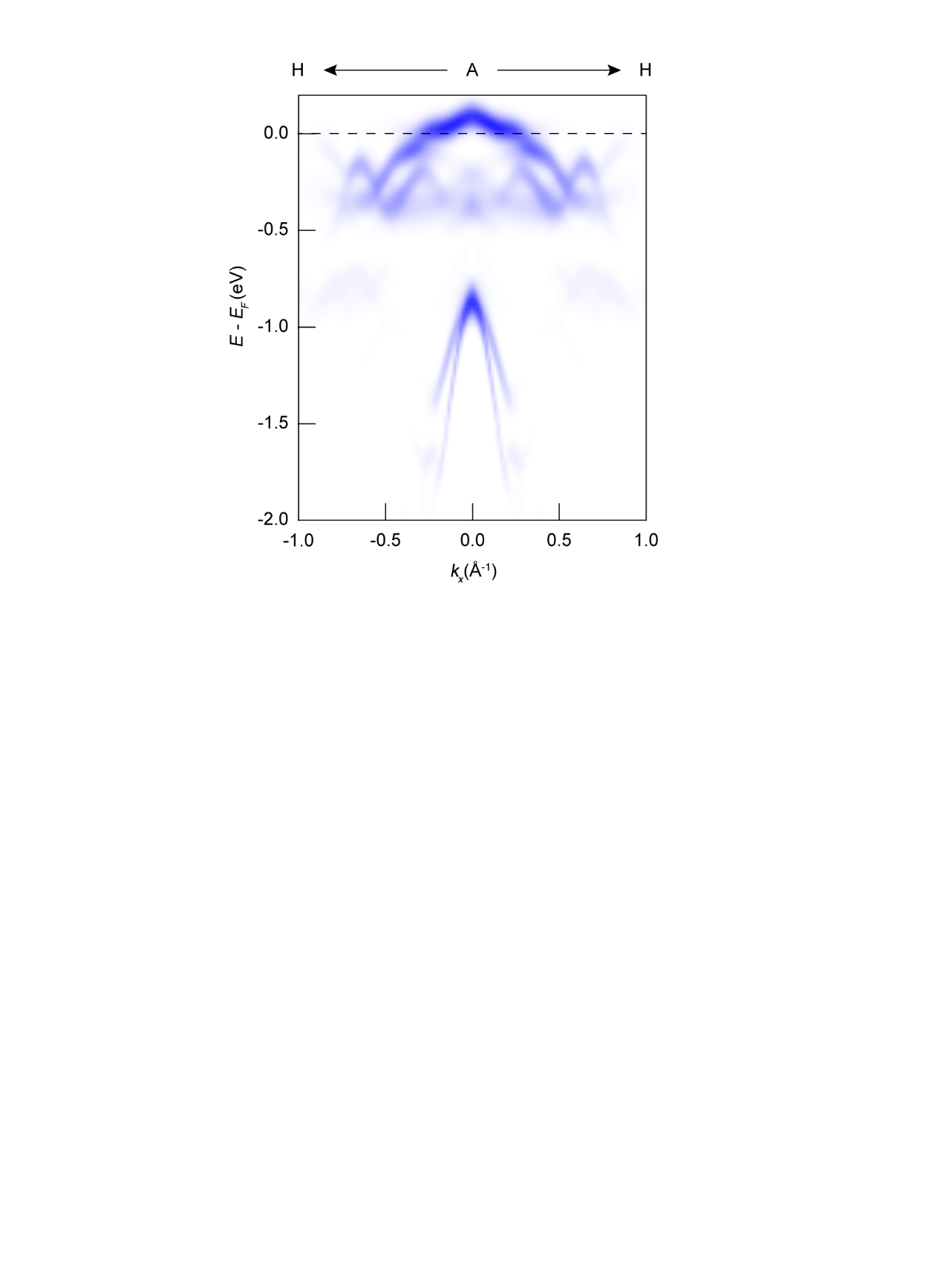


**Supplementary Fig. 11│DFT+*U*+*V* band structure of bulk 1T-TaSe_2_ along A-H direction of the undistorted atomic lattice Brillouin zone.** The first-principles calculations with self-consistent extended Hubbard interactions reproduce the hole-like metallic band and other features in the ARPES spectrum from Supplementary Fig. 5a (See Methods for details of the calculations).

1. **Orbital character of the V1 band and metallic band in DFT+*U*+*V* calculations**


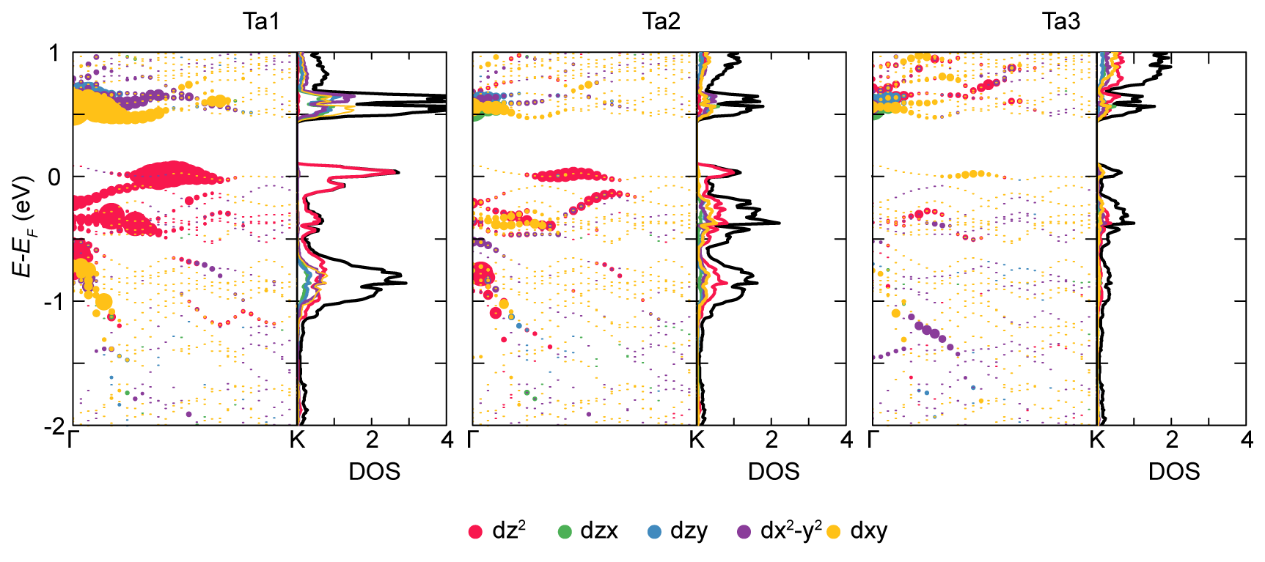


**Supplementary Fig. 12│Atomic and orbital decomposed DFT+*U*+*V* band structure of bulk 1T-TaSe_2_ along** $\boldsymbol{\Gamma-K}$**.** The contribution of $d_{z^{2}}$, $d_{zx}$, $d_{zy}$, $d_{{x^{2}-y}^{2}}$, and $d_{xy}$ of Ta atoms are shown in red, green, blue, purple, and orange, respectively. Ta atom at the center of star-of-David, its nearest neighbour and the next nearest neighbour are denoted by Ta1, Ta2 and Ta3, respectively. We project each orbital wave function onto the converged Kohn-Sham wave functions for the Kohn-Sham Hamiltonian with extended Hubbard interaction. The size of circle is proportional to the amplitude of projected orbital wave functions. We find that the contribution from Ta $d_{z^{2}}$ orbital dominates the Hubbard band and metallic band in bulk 1T-TaSe_2_.

1. **DFT+*U*+*V* calculation of the width of the metallic band in the nonmagnetic configuration**


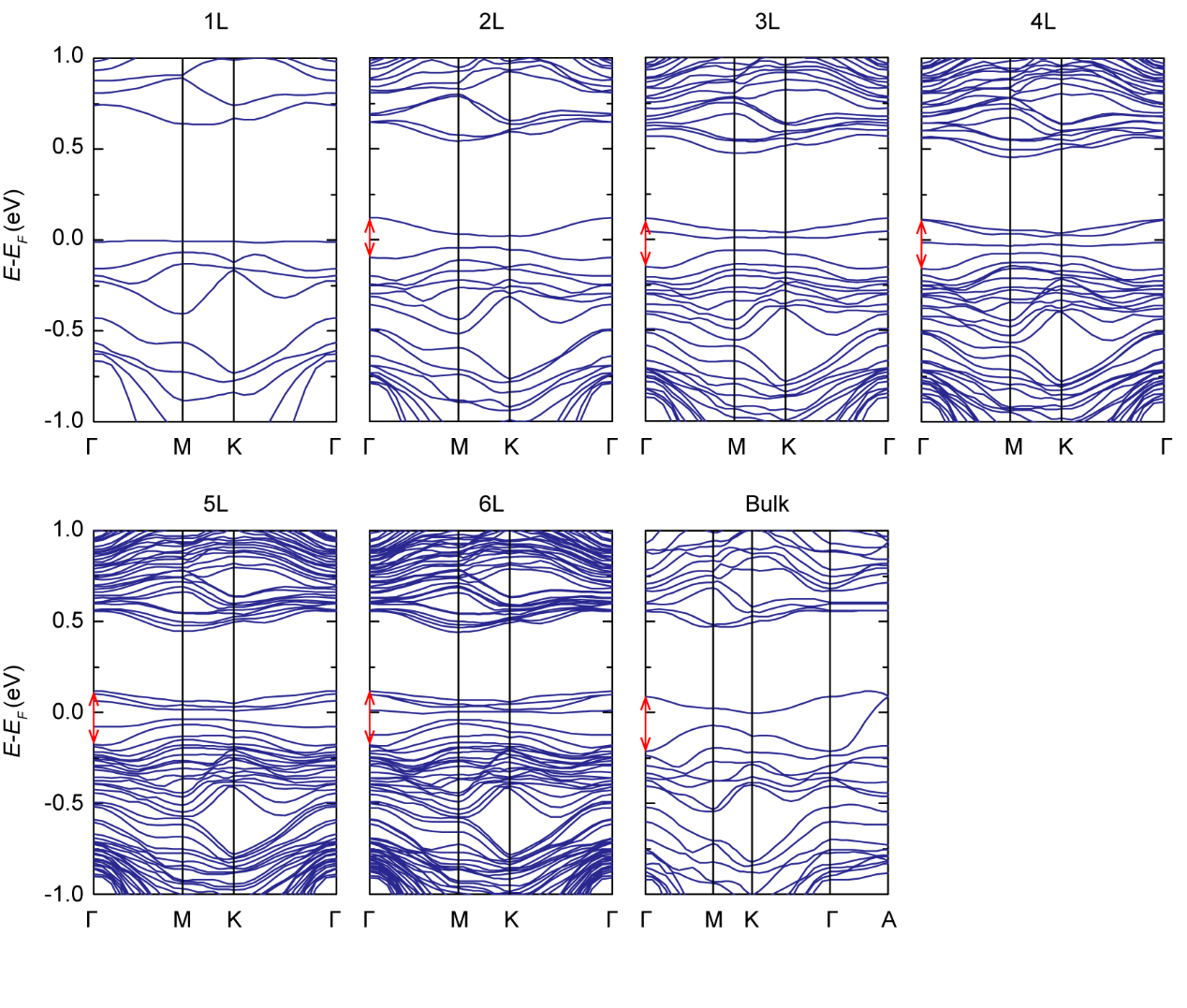
­­­

**Supplementary Fig. 13│DFT+*U*+*V* band structure of few-layer and bulk 1T-TaSe2 plotted in CDW superlattice Brillouin zone.** The single dispersive metallic band becomes $n$ bands near Fermi level in an $n$-layer 1T-TaSe_2_. We define the width of the metallic band as the combined width of the $n$ bands in the CDW superlattice Brillouin zone (red arrows). The bandwidth in monolayer 1T-TaSe_2_ is near zero, whereas thicker 1T-TaSe_2_ has a finite bandwidth as a result of interlayer coupling.

1. **Values of** $\boldsymbol{U}$ **and** $\boldsymbol{W}$ **in bulk and few-layer 1T-TaSe_2_**


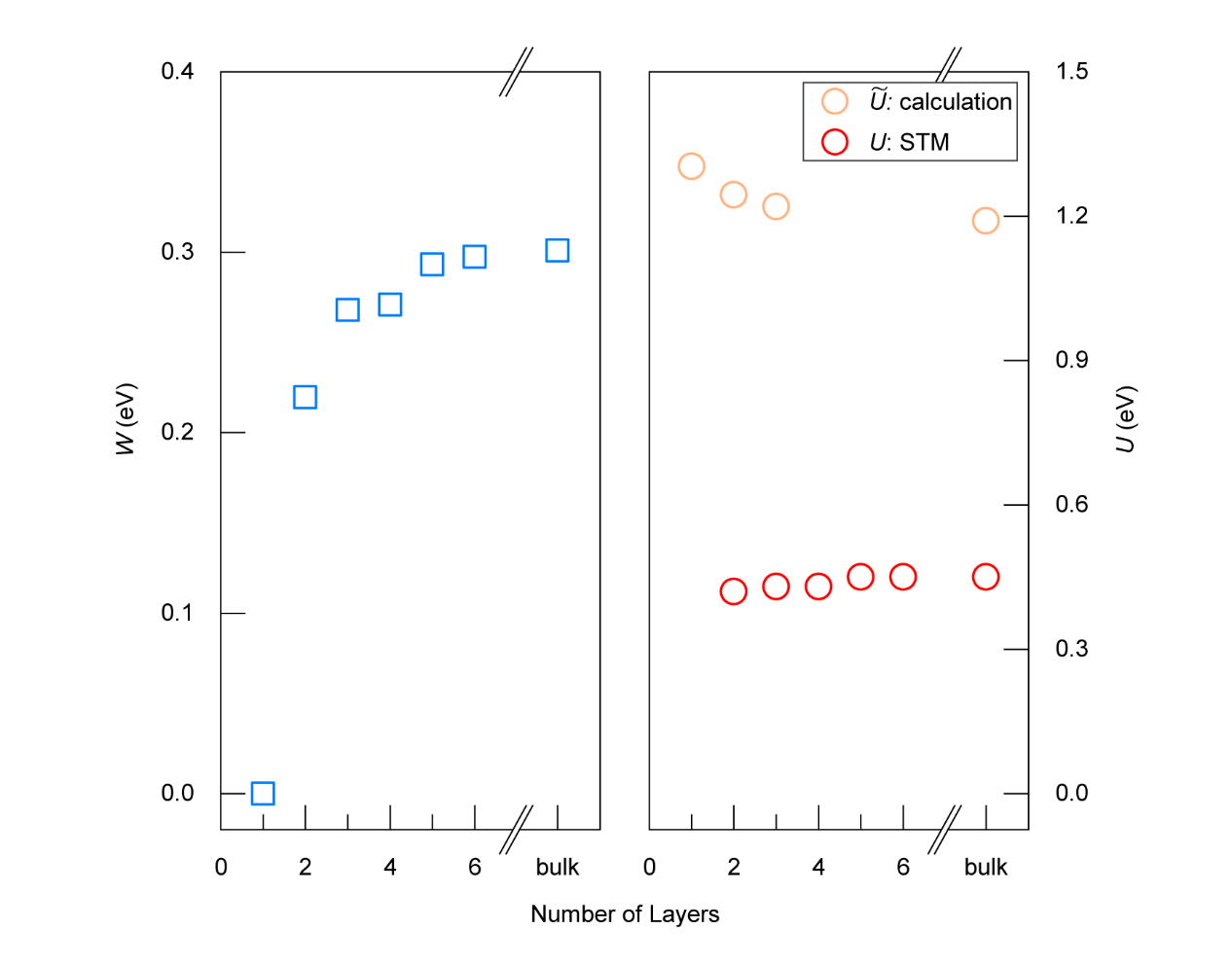


**Supplementary Fig. 14│Values of** $\boldsymbol{U}$ **and** $\boldsymbol{W}$ **obtained from experiment and self-consistent first-principles calculations.** The onsite Hubbard $U$ of the star-of-David cluster was extracted from the spectral gap in the differential conductance spectra in Fig. 2. Meanwhile, the onsite Coulomb energy of the Ta *d* orbital at the center of the star-of-David $\tilde{U}$ was calculated with the Agapito-Curtarolo-Buongiorno Nardeli pseudo-hybrid functional method [6, 12]. As the number of layers increases from monolayer to bulk, we found that the self-consistent $\tilde{U}$ value for the center Ta atom of the David star varies from $1.304 eV$ to $1.1904 eV$. Neither $U$ and $\tilde{U}$ exhibit strong thickness dependence. The bandwidth $W$, obtained from first-principles calculations in Supplementary Fig. 10, however, depends strongly on the sample thickness. We attribute the metal to Mott insulator transition to the suppression of $W$ (and consequently the $W/U$) in few-layer 1T-TaSe_2_. It is interesting to compare the situations in 1T-TaSe_2_ and 1T-TaS_2_, both of which have a star-of-David CDW ground state. Our calculations show that the Se $p$ orbital has a broader spatial distribution. The interlayer hopping in 1T-TaSe_2_ is therefore stronger, leading to a larger bandwidth than that in 1T-TaS_2_ [13]. We also note that the Se $p$ orbital in 1T-TaSe_2_ is located closer to the V1 band compared to the S $p$ orbital in 1T-TaS_2_ [14]. The smaller energy difference between Ta $d$ and Se $p$ orbitals leads to a stronger $p-d$ hybridization in 1T-TaSe_2_. So, even though both 1T-TaSe_2_ and 1T-TaSe_2_ on the verge of the transition between the metal and Mott insulator phase, bulk 1T-TaSe_2_ is metallic whereas bulk 1T-TaS_2_ is a Mott insulator [15].

1. **References**

1. Colonna S, Ronci F, Cricenti A *et al.* Mott phase at the surface of 1T-TaSe_2_ observed by scanning tunneling microscopy. *Phys Rev Lett*. 2005; **94**(3): 036405.

2. Qiao S, Li X, Wang N *et al.* Mottness collapse in 1T-TaS_2-x_Se_x_ transition-metal dichalcogenide: An interplay between localized and itinerant orbitals. *Phys Rev X*. 2017; **7**(4): 041054.

3. Chen Y, Ruan W, Wu M *et al.* Strong correlations and orbital texture in single-layer 1T-TaSe_2_. *Nat Phys*. 2020; **16**(2): 218-224.

4. Garrity KF, Bennett JW, Rabe KM *et al.* Pseudopotentials for high-throughput DFT calculations. *Computational Materials Science*. 2014; **81**: 446-452.

5. Hamada I. van der Waals density functional made accurate. *Phys Rev B*. 2014; **89**(12): 121103.

6. Lee S-H, Son Y-W. First-principles approach with a pseudohybrid density functional for extended Hubbard interactions. *Physical Review Research*. 2020; **2**(4): 043410.

7. Yang W, Jhi S-H, Lee S-H *et al.* Ab initio study of lattice dynamics of group IV semiconductors using pseudohybrid functionals for extended Hubbard interactions. *Phys Rev B*. 2021; **104**(10): 104313.

8. Yang W, Jang BG, Son Y-W *et al.* Lattice dynamical properties of antiferromagnetic oxides calculated using self-consistent extended Hubbard functional method. *Journal of Physics: Condensed Matter*. 2022; **34**(29): 295601.

9. Medeiros PVC, Stafström S, Björk J. Effects of extrinsic and intrinsic perturbations on the electronic structure of graphene: Retaining an effective primitive cell band structure by band unfolding. *Phys Rev B*. 2014; **89**(4): 041407.

10. Medeiros PVC, Tsirkin SS, Stafström S *et al.* Unfolding spinor wave functions and expectation values of general operators: Introducing the unfolding-density operator. *Phys Rev B*. 2015; **91**(4): 041116.

11. Lee S-H, Goh JS, Cho D. Origin of the insulating phase and first-order metal-insulator transition in 1T-TaS_2_. *Phys Rev Lett*. 2019; **122**(10): 106404.

12. Agapito LA, Curtarolo S, Buongiorno Nardelli M. Reformulation of DFT+U as a pseudohybrid Hubbard density functional for accelerated materials discovery. *Phys Rev X*. 2015; **5**(1): 011006.

13. Ritschel T, Trinckauf J, Koepernik K *et al.* Orbital textures and charge density waves in transition metal dichalcogenides. *Nat Phys*. 2015; **11**(4): 328-331.

14. Ang R, Miyata Y, Ieki E *et al.* Superconductivity and bandwidth-controlled Mott metal-insulator transition in 1T-TaS_2-x_Se_x_. *Phys Rev B*. 2013; **88**(11): 115145.

15. Inada R, Ōnuki Y, Tanuma S. Hall effect of 1T-TaS_2_ and 1T-TaSe_2_. *Physica B+C*. 1980; **99**(1): 188-192.
